# Why iPlay: The Relationships of Autistic and Schizotypal Traits With Patterns of Video Game Use

**DOI:** 10.3389/fpsyg.2022.767446

**Published:** 2022-02-23

**Authors:** Nancy Yang, Pete L. Hurd, Bernard J. Crespi

**Affiliations:** ^1^Department of Biological Sciences, Simon Fraser University, Burnaby, BC, Canada; ^2^Department of Psychology, Neuroscience and Mental Health Institute, University of Alberta, Edmonton, AB, Canada

**Keywords:** autism, schizotypy, video games (psychology), play, imagination, evolution, social cognition, technology

## Abstract

Video games are popular and ubiquitous aspects of human culture, but their relationships to psychological and neurophysiological traits have yet to be analyzed in social-evolutionary frameworks. We examined the relationships of video game usage, motivations, and preferences with autistic and schizotypal traits and two aspects of neurophysiology, reaction time and targeting time. Participants completed the Autism Quotient, Schizotypal Personality Questionnaire, a Video Game Usage Questionnaire, and two neurophysiological tasks. We tested in particular the hypotheses, motivated by theory and previous work, that: (1) participants with higher autism scores would play video games more, and participants with higher schizotypy scores would play video games less; and (2) autism and positive schizotypy would be associated with opposite patterns of video game use, preferences and motivations. Females, but not males, with higher autism scores played more video games, and exhibited evidence of relatively male-typical video game genre preferences and motivations. By contrast, positive schizotypy was associated with reduced video game use in both genders, for several measures of game use frequency. In line with previous findings, males played video game more than females did overall, preferred action video games, and exhibited faster reaction and targeting times. Females preferred Puzzle and Social Simulation games. Faster reaction and targeting times were associated with gaming motives related to skill development and building behavior. These findings show that gaming use and patterns reflect aspects of psychology, and gender, related to social cognition and imagination, as well as aspects of neurophysiology. More generally, the results suggest that video game use is notably affected by levels of autistic and schizotypal traits, and that video games may provide an evolutionarily novel medium for imaginative play in which immersive play experiences can be decoupled from social interaction.

## Introduction

Why do people play? Play is a nearly universal behavior among mammals (Burghardt, 2005), but only humans have the capacity for complex and social pretend play, and for developing the multiple orders of intentionality necessary for narrative production, theory-of-mind, and abstract thinking (Nowell, 2016). Play has also been postulated as a fundamental preparatory adaptation for higher-order adult social behaviors such as competition, reciprocity, and moral development (Bekoff, 2001; Bateson, 2005). There is also evidence that play may be considered a form of proto-creativity, which may underlie higher-order social cognitive processes including imagination, theory-of-mind, and abstract problem-solving (Vygotsky, 1967; Leslie, 1987). For example, in both object and pretend play, there is a transitive process where children spontaneously extract or conjure abstract properties of objects, manipulate or “play” with such constructs, then project them onto other entities. The transitive process of spontaneously conjuring abstract properties and manipulating them among unrelated objects is a hallmark of imagination, creativity, and divergent thinking. Physical play, such as rough-and-tumble play, has also been implicated in the development of social behaviors, such as dominance and cooperation (Smith and Boulton, 1990). Thus, play appears to be a key ontogenetic developmental phase in human social cognitive development.

How, then, does the current digitalization of the social world affect how people play? Given increased virtualization of the social world, video games have come to increasingly predominate the “iGen” (i.e., the generation of individuals born between 1995 and 2011, who grew up with smartphones and social media) play space (Twenge, 2017). As of 2021, there are nearly 227 million video game players in the U.S., with the median age at 31 years old (Entertainment Software Association [ESA], 2021), making video games one of the most popular pastimes enjoyed by people from all age groups. Annual video game sales in the U.S. have also grown from $25 billion in 2016 to $35 billion in 2018 (Entertainment Software Association [ESA], 2019), making it one of the most lucrative and fastest growing industries in the entertainment sector. The increasing accessibility and ubiquity of digital technology, such as mobile phones and tablets, have made it possible for most of the population to access games at their fingertips. The increasingly accessibility of video games has also made it possible for games to be incorporated into a diverse range of day-to-day activities, including but not limited to education, entertainment, rehabilitation, and therapy.

Despite the growing importance and popularity of video games in modern life, the relationships of video game culture with social and cognitive phenotypes remain little understood. To date, most research on video games had focused on specific aspects of neuropsychological traits to video game use, such as the relationship between aggression and violent video games (Scott, 1995), the use of video games in training faster reaction times (Dye et al., 2009), and the effects of Big Five personality traits on video game preferences (Peever et al., 2012). Such work is useful for elucidating the proximate mechanisms underlying video game use but does not capture the overarching questions of how and why video game usage is involved in the larger socio-evolutionary framework of play, social cognitive development, and imaginative culture. In other words, why do people play video games? Why is such play important? What social-cognitive, psychological, and neurophysiological traits are associated with video game usage, preferences, and motivations? And how do video games factor into the evolutionary functions of play in general?

Current evidence suggests that video game usage patterns are consistent with psychological profiles and behaviors that mirror real life motivations and preferences (Wang et al., 2019; Delhove and Greitemeyer, 2020). For example, self-perceived in-game aggression in the First Person Shooter game Overwatch is positively associated with trait aggression, Dark Tetrad traits, and negatively with empathy and agreeableness in players (Delhove and Greitemeyer, 2020). Multiple lines of evidence also suggest Big Five personality traits have a measurable impact in individual patterns of video game usage (Tabacchi et al., 2017; Wang et al., 2019; Yang et al., 2020). For example, extraversion has been positively associated with preference for party, music, and casual games, and negatively with fantasy role-playing, MMORPGs (massively multiplayer online role playing games), action role-playing, and strategy games (Peever et al., 2012). Similarly, conscientiousness has been associated with preferences for sport, racing, flight simulation, and fighting games (Peever et al., 2012), which suggests that athletic and action video game genres involve clear and identifiable goals and immediate reinforcement of achievements, and conscientiousness is associated with greater goal orientation (Colquitt and Simmering, 1998). Openness to experience, a personality trait linked with creativity and imagination (McCrae, 1987), has been associated with preferences for action-adventure and platformer games (Peever et al., 2012), both of which usually involve open exploration of virtual environments and creative puzzle-solving (e.g., Legend of Zelda, Super Mario). Indeed, openness to experience has also been associated with greater motivation to play video games for immersive experiences (Johnson and Gardner, 2010). Individuals with higher social orientation tend to play competitive or multiplayer games such as Call of Duty or World of Warcraft and individuals with high goal orientation prefer exploratory, intrinsically rewarding games such as Minecraft (Tondello and Nacke, 2019).

How, then, does the development of diverse social-psychological phenotypes correlate with general video usage? From the pattern described above, different typologies of video game players may be elucidated further, based on differential development in socio-cognitive traits and usage profiles.

Emerging evidence indicates that video games usage may reflect not only users’ psychological traits, but also neurophysiological skills such as reaction time (Dye et al., 2009; Deleuze et al., 2017; Gorbet and Sergio, 2018; Kowal et al., 2018; Torner et al., 2019), spatial visualization (Dorval and Pepin, 1986), multiple object tracking (Green and Bavelier, 2006), better cognitive flexibility in task-switching (Li et al., 2020) and probabilistic inferences on visual perceptual task (i.e., being able to quickly discriminate whether a display of randomly moving dots are moving toward the left or right) that are generalizable across modalities (Green et al., 2010). Furthermore, video game users also exhibit better hand-eye coordination skills than non-users (Griffith et al., 1983), but the question of whether video game usage improves hand-eye coordination times, or whether users with better hand-eye coordination play more video games (or both), is not yet resolved.

Considered together, these results support the view that video game usage patterns are associated with variation in some psychological traits and neurophysiological phenotypes. However, most studies have focused on action games, and no previous work has analyzed subclinical autistic and schizotypal traits (i.e., traits related to social cognition, neurodevelopment, and other phenotypes) together in relation to video game usage and preferences and their neurophysiological correlates. Such studies are important given the important roles of video games of diverse genres in human imaginative culture, and given that individuals with autism or schizotypy show some evidence of high rates of video game usage (Mazurek and Engelhardt, 2013a,b; Schimmenti et al., 2017; Choi et al., 2020; Menear and Ernest, 2020; Coutelle et al., 2021). This study thus analyzes how video game usage patterns, genre preferences, motivations, and reaction times are associated with measures of non-clinical autism and schizotypy.

Two studies suggest that psychological variations related to the autism spectrum and the schizophrenia spectrum, including positive schizotypy, may mediate aspects of video game use. First, Wendt et al. (2019) reported a positive genetic correlation of computer game use with autism risk, and a negative genetic correlation of computer game use with schizophrenia risk. These findings suggest that autism and schizophrenia or schizotypy may show opposite patterns of association with video game use in non-clinical populations, for reasons that have yet to be investigated.

Second, according to the diametric model of social brain disorders (Crespi and Badcock, 2008; Crespi and Go, 2015; Crespi, 2016, 2019, 2020), autism and psychosis can be conceptualized as polar ends of a continuum of social cognition development, with normality at its center. Thus, autism is characterized by low mentalistic thought (i.e., social cognition) and high mechanistic thought (i.e., non-social cognition), and psychotic-affective conditions, such as schizotypy, are characterized by high mentalistic and low mechanistic thought (Crespi and Badcock, 2008). Given that childhood play can be categorized in non-social (i.e., object) and social (i.e., pretend or imaginary) domains, video game usage patterns may be expected to co-vary according to the socio-cognitive traits in autistic and schizotypal spectra.

Mechanistic thought is characterized by a cognitive style specialized for recognizing patterns in rule-based systems (i.e., “if p, then q”) (Baron-Cohen et al., 2009). In autism, a systemizing cognitive style is applicable to a broad swath of domains such as collectibles (i.e., distinguishing between types of objects and collecting them), motoric (i.e., throwing a Frisbee), mechanical (i.e., taking apart objects and reassembling them), spatial (i.e., fixed interests with routes), action sequences (i.e., analyzing dance techniques), and numerical traits (i.e., solving math or logical problems) (Baron-Cohen et al., 2009)—all of which are conducive for puzzles, action, platformer, sports, and construction simulation types of video games where extensive strategizing of rule-based systems is involved. Indeed, previous studies have found that individuals with autism prefer gaming genres such as Action, Platformer, and Shooter (Mazurek and Engelhardt, 2013b; Kuo et al., 2014), all of which challenge the player’s coordination, and speed in rule-based gameplay. A strong systemizing style can also involve especially high attention to details and fast sensory processing (Baron-Cohen et al., 2009), which may facilitate higher video game usage by enhancing attentional focus, reaction times, and hand-eye coordination.

Furthermore, autistic traits have been associated with higher video game usage (Mazurek and Engelhardt, 2013b), and problematic video game usage (i.e., symptoms of clinical addiction to video games) (Liu et al., 2017; Coutelle et al., 2021; Craig et al., 2021; Murray et al., 2021). Compared to neurotypicals, boys and male adolescents with ASD also play video games for longer times, prefer to play alone, and play less frequently in multiplayer mode (Paulus et al., 2019). Taken together, autistic traits may thus involve higher video game usage, as well as increased preferences for puzzle, action, platformer, strategy, racing, sports, idle, and construction and management simulation games. Given the positive association of action video games with autistic traits, we also expect that autistic traits in non-clinical individuals may also be positively associated with increased neurophysiological skills such as faster reaction and hand-eye coordination times.

In contrast to autism, positive schizotypy can be characterized by hyper-mentalistic traits such as fantasy-proneness, ideas of reference (i.e., illusory social references to self), paranoia (e.g., illusory eye contact and fear), and increased imagination (Crespi and Badcock, 2008; Crespi et al., 2016). All of these traits may be expected to increase preferences for fantasy or fantasy-based role-playing video games that are extensions of pretend play. In contrast to autism, which is known for its singular focus on select topics of special interests (Casey et al., 1993), schizotypy is associated with information processing impairments such as poor sustained attention (Lenzenweger et al., 1991), deficits in sensorimotor gating (i.e., inability to filter out irrelevant stimuli from the environment) (Cadenhead et al., 2000; Park et al., 2015), and deficits in smooth eye pursuits (Lenzenweger and O’Driscoll, 2006)—all traits that may make video games challenging, as they commonly involve detecting and tracking sudden and fast-moving objects and distractors while multi-tasking (Hubert-Wallander et al., 2011).

Positive schizotypal traits, in particular, have been associated with slower reaction time during conditions of high perceptual load (Lenzenweger, 2001) and deficits in predicting targeting hand movements (Asai et al., 2008), both of which would impede video gameplay, particular action genres, as a large component of gameplay involve fast decision-making and quick executions of hand-eye movements via controller use. Thus, positive schizotypal traits may be associated with decreased video game usage, as well as a preference for fantasy or fantasy-based role-playing video games, which tend to be slower-paced and emphasize user-directed exploratory behavior rather than time-intensive tasks.

Gender differences in play have been well-documented in the literature, extending into video game usage and preferences. Male play is more physically aggressive (Ostrov and Keating, 2004) and “thing-oriented,” with increased interests in objects and functions of objects such as construction and transportation (Servin et al., 1999). In comparison, female play tends to be more socially directed and involve fantasy-role playing, such as play parenting (i.e., doll playing), a sex difference that has been observed in primates (Pryce, 1995), and pretend play (i.e., toy tea-sets) (Servin et al., 1999). Preliminary lines of evidence suggest that sex differences in childhood play may also extend to video game usage. For example, college-aged males prefer more physical aggressive video game genres such as action, racing, and sports (Greenberg et al., 2010). In contrast, females prefer to prefer more “traditional” games such as puzzles, cards, classic arcade games, or board games (Greenberg et al., 2010), all of which are less physically aggressive. Thus, sex differences in patterns of video game usage, genre preferences, and motivations might be expected to reflect general sex differences in childhood play.

Based on the reasons outlined above, the following predictions can be made:

Hypothesis 1: Gender differences in video game usage, genre preferences, and motivations will vary in the following ways: (1) Males will report greater video game usage than females, (2) Males will prefer mechanistic-oriented video games such as Action, Platformer, Strategy, Racing, Sports, RPG, and Construction and Management Simulation games while females will prefer Puzzle and Social Simulation video games, (3) Males will report greater motivation for Skill Development and Customization games and females will report greater motivations of Social Interaction, a Way to Fantasize, and a Way to Escape on the Video Game Usage Questionnaire used here.

Hypothesis 2: Autistic traits will be associated with increased video game usage. Total and positive schizotypal traits, as quantified by total score on the SPQ-BR and scores on the SPQ Cognitive-Perceptual subscale, will be negatively associated with frequency of video game usage.

Hypothesis 3: Autistic traits will be positively associated with preferences for relatively rule-based video game genres including Puzzle, Action, Platformer, Strategy, Racing, Sports, Idle, and Construction and Management Simulation games. Total and positive schizotypal traits will be positively associated with preferences for Fantasy, Role Playing, and Social Simulation video games.

Hypothesis 4: Autistic traits will be positively associated with mechanistic motivations such as Skill Development and Customization on the Video Game Usage Questionnaire. Total and positive schizotypal traits will be positively associated with mentalistic motivations such as Social Interaction, Way to Fantasize, and Way to Escape on the Video Game Usage Questionnaire used here.

Hypothesis 5: Autistic traits will be associated with increased neurophysiological skills, such as faster reaction time and hand-eye coordination time. Conversely, total and positive schizotypal traits will be associated with slower reaction and hand-eye coordination times.

Hypothesis 6: Increased total video game usage will be predicted by higher reaction and hand-eye coordination time. In turn, slower reaction and hand-eye coordination time will predict lower video game usage.

The predictions of these hypotheses were tested using data on measures of autism spectrum and schizotypy spectrum cognition in a non-clinical population, and data on reaction times and targeting times, two measures of neurophysiological performance that may be related to video game use and performance.

## Materials and Methods

### Participants

A total of 351 participants (207 females, 144 males, mean age = 20.20 ± 3.04 years old) were included in the study. The participants were recruited from various electronic mailing lists (i.e., university student lists and newsletters) and the Simon Fraser University Undergraduate Psychology Research Pool. The following study description was circulated as the recruitment email: “Do you play video games? Or do you not play video games? Either way, we want to hear from you! We are doing an online study on the relationship between psychological traits and video game usage. You will receive a $10 gift card for participation. For more info please contact nancy_yang_2@sfu.ca.” Participants were reimbursed with either a course credit or a $10 electronic gift card for their time. All participants provided informed consent before participating in this study. Data collection took place during the Spring 2021 semester (January–April 2021). The study was approved by the Simon Fraser University Research Ethics Board (Study Permit 2020s0503).

### Psychological Questionnaires

The Autism Spectrum Quotient (AQ) was used to assess individual variations in autistic traits (Baron-Cohen et al., 2001). The AQ comprises 50 questions assessing five different domains: (i) social skills, (ii) attention switching, (iii) attention to detail, (iv) communication, (v) imagination. Responses were scored in a 4-point Likert-scale format from “definitely agree” to “definitely disagree.” Participants score one point when they report a trait that is consistent with the autism spectrum, for a possible scoring range of 0–50. Higher scores on the Social Skills and Imagination subscale represent lower social skills and lower social imagination.

Schizotypal traits were quantified with Schizotypal Personality Questionnaire-Brief Revised (SPQ-BR) (Cohen et al., 2010). The SPQ-BR includes 32 items in a 5-point Likert scale format with response choices ranging from “strongly disagree” to “strongly agree.” Possible scores range from 0 to 160, with higher scores indicative of higher levels of schizotypy. The SPQ-BR is clustered into three superordinate factors: Cognitive-Perceptual, Interpersonal, and Disorganized schizotypy—which map onto the positive, negative, and disorganized dimensions of schizophrenia, respectively (Andreasen et al., 1995). The Cognitive-Perceptual (positive) schizotypy scale (referred to here as SPQ-CogPer) consists of three subscales: Magical Thinking, Unusual Perceptions, and Ideas of Reference. The Interpersonal schizotypy factor includes Social Anxiety and Constricted Affect, and the Disorganized factor includes Eccentric Behavior and Odd Speech.

A Video Game Usage Questionnaire was adapted from the Gaming Attitudes, Motives, and Experiences Scales (GAMES) (Hilgard et al., 2013). The questionnaire has six main sections to assess patterns of video game usage, as well a short demographic section that asks participants to report their age and gender (ex: male, female, other, prefer not to answer). First, participants were asked the amount of weekday and weekend hours spent on video games, as well as self-evaluations of video game usage. Participants are also asked the titles of the video games that they play, preferred types of video game genres, motivations for gaming (i.e., Social Interactions, Stress Relief, Skill Development, Adrenaline Rush, A Place to Escape, A Way to Fantasize, Customization), self-evaluations of video game performance, as well as preferred type of device for playing video games. For game titles, participants were given a list of popular video games to choose from, as well as write their own response in an open-ended “Other” answer box. For video game genres, participants were given the following list to choose from, as well as brief examples of what each genre entails. The list of video game genres was as follow: Puzzle games (candy crush, portal), Action games (first-person shooter like Doom, Call of Duty), Platformer games (side scrolling games like Mario, Sonic), RPG (action-based role-playing games, Darkest Dungeon, Skyrim, Kingdom-come: Deliverance), Strategy (turn based or real time—Xcom, Civilization, Total Warhammer), Sports games (tennis, golf), Construction and management simulation (Minecraft), Social Simulation (Animal Crossing), Idle games (Cookie click, idle heros), and Other (open ended response where participants could write their own). Given that most video games overlap somewhat in their genres, we hereby differentiate action-oriented role-playing games and fantasy-based role-playing games by categorizing the former as “RPG,” as per popular usage (Rehbein et al., 2016), and the latter as “Social Simulation” to better emphasize differences between these two genres. We excluded responses entered in the “Other” video game genre question item as the responses were too few to reach statistical power.

The frequency of video game usage was quantified via participant self-evaluations on this questionnaire. There were five questions pertaining to video game usage. First, participants were asked on the following: (1) Self report usage: “What is your video game usage like?” on a 5-point Likert scale where 1 was “Very Often” and 5 was “Very rarely or not at all,” (2) Self report frequency: “How frequent do you play video games? Check one.” Participants had the option of selecting from “Daily,” “2–3 times a week,” “weekly,” “2–3 times a month,” “monthly,” “less than monthly,” and “never,” (3) Self report spare time: “What proportion of your spare time is spent on video games?” on a 5-point Likert scale where 1 was “Almost all of none of my spare time” and 5 “Almost all of my spare time.” For self-reported frequency, responses were scored as “1” if participants answered “Never” and “7” for “Daily.” Participants were also asked to enter the number of hours spent on video games for an average weekday and weekend day.

### Experimental Procedures

Participants were first given the battery of questionnaires to complete, in the following order: Video Game Usage Questionnaire, AQ, and SPQ-BR. Afterward, participants completed two computer tasks (1) Fitts Law Task to quantify targeting time, and (2) Simple Choice Task to quantify reaction time. Both computer tasks were administered via https://www.psytoolkit.org/ (Stoet, 2010, 2017), a toolkit for online cognitive-psychological experiments and surveys^1^. The questionnaires were administered via SurveyMonkey^2^. The computer tasks are described below.

Fitts’ Law Task (20 Trials in total) (Fitts, 1954): There were 20 trials in total. On each trial, the participant was presented with a yellow square on the left upper side of a black screen. The participant was instructed to click on the yellow square with their mouse cursor. After the yellow square was clicked on, a red rectangle, of a randomized size, appeared on a random place on the screen. The participant was then instructed to move their mouse cursor to the red rectangle as quickly as possible, for a total of 20 times. When participant completed the Fitts’s Law Task, they were presented with a feedback screen that said: “Great job! Press the space bar to continue.” Then the participants were presented with a short summary of the task objectives: “Fitts’s Law. The time it takes you to move the cursor on the red rectangle depends on the size and the distance. Press space bar to exit.” After completion of the Fitts’ Law Task, the participant moved onto the Simple Choice Task.

Simple Choice Task (8 Training Trials, 20 Experimental Trials) (Deary et al., 2011): For this task there were 28 trials in total, with 8 practice and 20 experimental trials. On each trial, a black cross appeared on a white square on the computer screen. Participants were instructed to press the space bar as soon as possible when they see a black cross appearing in the white box. The time when the cross appeared in the white box varied from trial to trial. No feedback on performance was given for either computer tasks.

### Analyses

Analyses were conducted in R (v4.0.5) (R Core Team, 2021) using functions in the base statistical package. Linear regressions were conducted using the lm() function while logistic regressions were conducted using the glm() function with binomial response family. Stepwise elimination of independent variables, for stepwise regressions, was accomplished using the step() function. Two extreme outliers were removed from the reaction time analysis, due to them being over eight standard deviations from the other reaction time scores. To assess the relationship between the frequency of video game use and the thirteen AQ and SPQ subscales we conducted multiple regressions for each of the five measures of videogame use. We also conducted separate analyses of covariance (ANCOVAs) for each of these dependent variables, using each of the AQ and SPQ subscale scores as independent variables, and gender as a cofactor. To assess the relationship between the frequency of video game use and the composite, we conducted ANCOVAs as for the subscales and included interaction terms along with the gender cofactor. Correlations between reaction time and targeting score were assessed using a linear model analysis of covariance using the targeting score as the outcome variable and reaction time as independent variable, with gender as a covariate.

Effects of reaction time and targeting scores on each of the five measures of video game use were analyzed with ANCOVAs using each of the game use variables as an outcome and either reaction time or targeting time score as the independent variable, and gender included as a covariate, and without an interaction term. To analyze the effects of the AQ and SPQ subscales, we performed backward stepwise regressions starting with each all of the AQ and SPQ subscales and gender as independent variables for each of the five use measure outcome variables. The least informative independent variable was iteratively dropped from the model until dropping a variable led to a less efficient model, as assessed using the Akaike information criterion.

Tests for association between reaction time, and targeting scores, on the type of games played were conducted using logistic regressions. Each of the game types was modeled with a separate analysis, with type of game played as a Boolean outcome; the independent variables were either reaction time, or targeting score, each of which also included gender as a covariate, without interaction terms. Tests for the association between reaction time and targeting scores and each of the different types of motivation to play games were conducted in the same manner, with one logistic regression of the motivational type as a Boolean outcome variable against either reaction time or targeting with gender included as a covariate.

To test for associations of AQ and SPQ scales with the type of games played, logistic regressions were conducted using general linear models with binomial response families. Each of the game types was modeled with a separate analysis, with type of game played as a Boolean outcome, and all subscales were entered as independent variables along with gender. No interaction terms were entered. A backward stepwise method was used to successively drop the least informative independent variable, until dropping a variable led to a less efficient model, as assessed using the Akaike information criterion. Tests for association between the AQ and SPQ subscales and the motivations to play games were conducted in the same manner, with a separate stepwise elimination of subscales until reaching the most efficient model for each of the different motivation types. Simple bivariate analyses (e.g., *t*-tests, product-moment correlations) were conducted for some analyses, in addition to ANCOVAs and multiple regression analyses, for representation and depiction of the results for each relevant test, since these analyses make fewer assumptions.

## Results

### Gender Differences in Autism Spectrum Quotient, Schizotypal Personality Questionnaire, and Patterns of Video Game Usage

Females scored higher than males on SPQ-Ideas of Reference, SPQ-Social Anxiety, SPQ-Magical Thinking, SPQ-Odd Speech, SPQ-Cognitive-Perceptual, SPQ-Disorganized, and SPQ-Total (Supplementary Tables 1, 2). There were no outliers in the AQ-total or SPQ-total scores (Supplementary Figures 1, 2).

Males scored higher than females on all the variables that quantified video game usage (Supplementary Tables 1, 2). Males also demonstrated faster reaction and targeting time on the Simple Choice Task and Fitts’ Law Task than females, respectively (Supplementary Tables 1, 2).

Females preferred Puzzle and Social Simulation games more than males did. By contrast, males preferred Action, RPG, Strategy, and Sports games (Supplementary Tables 1, 2). Males were more motivated by Social Interaction, Skill Development, Adrenaline Rush, and Fantasy reasons compared to females. No gender differences were detected for the motivations Stress Relief or Escape. Females were more motivated by Customization than males.

### Patterns of Video Game Use in Relation to Autism Spectrum Quotient-Total, Schizotypal Personality Questionnaire-Total, and Schizotypal Personality Questionnaire-CogPer

**FIGURE 1:**
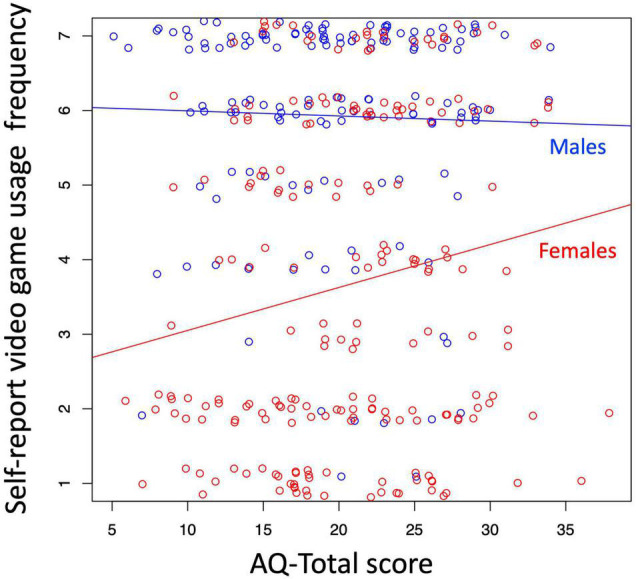
Relationship between self-reported frequency and AQ scores for females (red) and males (blue).

By product-moment correlations, higher AQ-total scores were significantly associated with higher video game use for the following usage measures: weekend hours, self-reported frequency, and self-reported usage, in females only (Table 1). Higher SPQ-CogPer scores were associated with reduced weekday and weekend hours, as well as decreased self-reported frequency and self-reported spare time spent on video games, when both genders were pooled together. In females only, higher SPQ-CogPer scores were also associated with decreased self-reported spare time video games usage. No statistically significant relationships were found for SPQ-Total. Higher AQ-Total scores were positively associated with higher self-reported frequency of video game usage in females, while male video game usage was relatively high across the entire range of AQ-Total scores (Figure 1).

**TABLE 1:** Pearson Correlations of AQ-total, SPQ-total, and SPQ-CogPer scores with Measures of Video Game Usage.

Video game usage					
	Weekday time (h)	Weekend time (h)	Self-report frequency	Self-report usage	Self-report spare time
**Females (*N* = 207)**					
AQ: Total	0.13	0.21**	0.17**	−0.20**	0.11
SPQ: CogPer	–0.05	–0.06	–0.12	–0.04	−0.17*
SPQ: Total	–0.02	0.05	–0.01	–0.10	–0.03
**Males (*N* = 144)**					
AQ: Total	–0.02	0.04	–0.03	0.07	0.05
SPQ: CogPer	–0.04	–0.03	–0.10	0.02	–0.04
SPQ: Total	0.03	0.06	–0.02	–0.10	0.11
**Both Genders (*N* = 351)**					
AQ: Total	0.02	0.08	0.04	–0.06	0.04
SPQ: CogPer	−0.13**	−0.14**	−0.20***	0.06	−0.18***
SPQ: Total	–0.05	–0.02	–0.09	–0.04	–0.04

Note that the scale for “Self report usage” is directionally opposite to the other scales. *Denotes significance at < 0.05. **Denotes significance at < 0.01. ***Denotes significance at < 0.001. Boldface values indicate that relationships were statistically significant.

ANCOVAs revealed that higher AQ-total was associated with greater weekend time, higher self-reported frequency, and higher self-reported usage of video game usage with an interaction effect indicating that the effects were restricted to females (Supplementary Table 3 and Figure 1). Higher scores on SPQ-CogPer were associated with lower self-reported frequency and lower self-reported spare time, with no gender interaction effect (Supplementary Table 3).

Both forms of analyses showed that higher AQ-total scores were associated with greater weekend time, self-reported frequency, and self-reported usage, in females only. Higher scores on SPQ-CogPer were also associated with lower self-reported spare time in females only and in both genders pooled together. Higher scores on SPQ-CogPer were also associated with lower self-reported frequency scores in both genders together.

### Patterns of Video Game Use in Relation to Autism Spectrum Quotient and Schizotypal Personality Questionnaire Subscales

By product-moment correlations, in females only, scores on AQ-Attention to Detail were associated with greater weekday hours spent on video games (Supplementary Table 4). AQ-total was not associated with any of the video game usage variables when males were analyzed separately or with the genders pooled together. In females only, AQ-Social Skills scores were positively associated with greater weekend time, self-reported frequency, and self-reported usage spent on video games. In females only, scores on AQ-Imagination scores were associated with greater weekend time and self-reported frequency spent on video games.

In females only, higher SPQ-Magical Thinking was associated with decreased self-reported spare time, while SPQ-Social Anxiety and SPQ-Disorganization were associated with greater weekend time on video games. In females only, SPQ-Eccentric Behavior scores were also positively associated with greater weekend time and self-report spare time on video games, with the relationship marginally non-significant (*p* = 0.06).

In males only, higher SPQ-Magical Thinking was associated with greater self-reported usage and spare time spent on video games. Higher SPQ-Eccentric Behavior was associated with greater self-reported usage and spare time on video games. In males, higher SPQ-Disorganization was marginally non-significantly associated with greater weekend time (*p* = 0.06), and significantly associated with greater self-reported usage, and self-report spare time on video games.

When both genders were pooled together, higher SPQ-Magical Thinking was associated with decreased weekday, weekend, self-reported frequency, self-reported usage, and self-reported spare time on video games. Higher SPQ-Ideas of Reference scores were associated with decreased self-reported frequency on video games. Higher SPQ-Unusual Perception was associated with decreased weekday hours on video games. Higher SPQ-Eccentric Behavior scores were associated with greater weekend time, self-reported usage, and self-reported spare time on video games.

By ANCOVAs, higher SPQ-Eccentric scores and SPQ-Magical Thinking were associated with higher and lower self-report spare time spent on video games, respectively (Supplementary Table 5). The combined results from both analysis revealed that higher SPQ-Magical Thinking was associated with lower self-reported spare time spent on video games in females, in males, and in both genders combined. Higher SPQ-Eccentric was also associated with greater self-reported spare time spent on video games in males only, and in both genders combined.

### Video Game Genre Preferences in Relation to Autism Spectrum Quotient-Total, Schizotypal Personality Questionnaire-Total and Schizotypal Personality Questionnaire-CogPer Scores

By *t*-tests, higher AQ scores were associated with increased preference for Construction type games and higher SPQ scores were associated with preferences for Social Simulation, when both genders were pooled together (Supplementary Table 6). Higher AQ-total scores were also associated with decreased preference for Sports, and higher SPQ-total scores were also associated with preference for Puzzle and Racing. Higher SPQ-CogPer scores were associated with increased preference for Puzzle and Racing games, and decreased preferences for RPG and Construction type games.

When males were analyzed separately, higher AQ-total scores were associated with decreased preference for Platformer and Sport games. Higher SPQ-total and SPQ-CogPer scores were associated with increased preference for Racing games in males. When females were analyzed separately, higher AQ-total scores were associated with increased preference for RPG and Construction games. Higher SPQ-total and SPQ-CogPer scores were associated with increased preference for Puzzle games in females.

Backward stepwise regression, conducted to test the effects of AQ-total, SPQ-total, and SPQ-CogPer on each video game genre while taking account of gender differences, showed that males reported higher preference for Action and Strategy games (Supplementary Table 7). Preferences for Action and Strategy video games were not associated with AQ-total, SPQ-total, or SPQ-CogPer. Higher AQ-total scores were associated with preference for RPG and Construction games in females. Higher SPQ-CogPer scores and higher SPQ-Total were associated with higher preference for Puzzle games. Both sets of results indicated that females with higher AQ-total scores reported a higher preference for RPG and Construction games.

### Video Game Genre Preferences in Relation to Autism Spectrum Quotient and Schizotypal Personality Questionnaire Subscales

Preference for Puzzle games was associated with higher AQ-Imagination, higher SPQ-Eccentric Behaviors, and in females. Preference for Action games was associated with higher SPQ-Eccentric Behaviors in males (Supplementary Table 7). Preference for Platformer was associated with higher SPQ-Eccentric Behaviors. Preference for RPG was associated with higher AQ-Social Skills, lower SPQ-Ideas of Reference, and higher SPQ-Eccentric, and in males. Preference for Strategy was associated with higher SPQ-Eccentric Behaviors and in males. Preference for Sports was associated with lower AQ-Communication and in males. Preference for Construction was associated with higher AQ-Social Skills, marginally with lower SPQ-Ideas of Reference, lower SPQ-Magical Thinking, and higher SPQ-Odd Speech. Preference for Social Simulation was detected in females, but no relationship to either AQ or SPQ subscales. Preference for Idle games was marginally associated with AQ-Attention Switch.

### Video Game Motivations in Relation to Autism Spectrum Quotient -Total, Schizotypal Personality Questionnaire-Total and Schizotypal Personality Questionnaire-CogPer

By *t*-tests, females with higher AQ-Total scores were motivated by Stress Relief, Fantasy, and Customization (Supplementary Table 8). Males with higher SPQ-CogPer scores were motivated by Stress Relief and Skill Development. Males with higher SPQ-total scores were motivated by Escape and Customization. Males with higher AQ-Total scores were more motivated to play video games for Customization.

When both genders were pooled together, higher AQ-Total scores were associated with motivations of Skill Development, Fantasy, Customization, and Stress Relief. Higher SPQ-Total scores were also associated with the motivation of Stress Relief.

By multiple regression analyses, Social Interaction was associated with decreased AQ-Social Skills, increased AQ-Attention to Detail, increased SPQ-Social Anxiety, and decreased SPQ-Magical Thinking (Supplementary Table 9). Stress Relief was associated with increased AQ-Social Skills and increased SPQ-Unusual Perception. Skill Development was associated with increased AQ-Attention to Detail, increased SPQ-Idea of Reference, and increased SPQ-Social Anxiety. Adrenaline Rush was associated with lower AQ-Attention Switch, higher AQ-Attention to Detail, higher AQ-Communication, and higher AQ-Imagination. Escape was marginally associated with higher SPQ-Constricted Affect, higher SPQ-Eccentric Behavior, and marginally associated with SPQ-Magical Thinking. Fantasy was associated with higher AQ-Social Skills and higher SPQ-Magical Thinking. Customization was associated with higher AQ-Attention to Detail and higher AQ-Communication. Higher AQ-Total was associated with greater motivations of Stress Relief, Fantasy, and marginally with Customization. No significant relationships were observed between SPQ-Total, SPQ-CogPer, and any of the motivations.

### Reaction and Targeting Times in Relation to Autism Spectrum Quotient -Total, Schizotypal Personality Questionnaire-Total, Schizotypal Personality Questionnaire-CogPer, and Gender

By product-moment correlations, in females only, faster reaction times were associated with higher AQ-Total and SPQ-Total scores (Supplementary Table 10). There was also a marginally non-significant trend of association of higher AQ-Total scores with faster targeting time in females (*p* = 0.06). No other statistically significant relationships were detected in males or in both genders pooled together.

### Reaction and Targeting Times in Relation to Autism Spectrum Quotient and Schizotypal Personality Questionnaire Subscale Scores, and Gender

Faster reaction times were associated with higher SPQ-Eccentric Behavior scores in both genders pooled together and in females. In females only, faster reaction times were associated with higher SPQ-Social Anxiety, SPQ-Disorganization AQ-Social Skills, and AQ-Attention to Detail. Higher SPQ-Magical Thinking scores were associated with slower reaction time in both genders pooled together and in males only (Supplementary Table 10).

Higher AQ-Social Skills and SPQ-Social Anxiety scores were associated with faster targeting times in females. Higher SPQ-Magical Thinking scores were associated with slower targeting times in both genders pooled together, in males, and in females. Higher SPQ-Unusual Perception scores were associated with slower targeting times in both genders pooled together. Faster targeting time associated SPQ-Interpersonal in males. Higher SPQ-CogPer scores were associated with slower targeting time in both genders and in males.

By multiple regression analyses, reaction times were significantly positively correlated with targeting times, with males being faster for both relative to females. No AQ or SPQ subscales were significantly associated with faster reaction times. Higher SPQ-Magical Thinking scores were associated with slower targeting times. These results are summarized in Supplementary Table 11. By both forms of analyses, males exhibited faster reaction and targeting times than females, and higher SPQ-Magical Thinking was associated with slower targeting times.

### Relationships of Reaction and Targeting Times With Video Game Usage, Preferences and Motivations

By product-moment correlations, faster targeting times were associated with increased self-reported spare time, weekend hours, weekday hours, greater self-reported video game usage and frequency in both genders pooled together, males only, and females only (Supplementary Table 12). Faster reaction times were also associated with greater self-reported of spare time, weekend hours, weekday hours, greater self-reported usage, and greater self-reported frequency of video game usage in both genders pooled together and in males (Supplementary Table 8).

By stepwise regression analyses, higher self-reported video game usage was associated with higher AQ-Attention to Detail, higher SPQ-Eccentric, faster targeting times, and being male. Higher self-report frequency of video game usage was associated with faster targeting times, and being male. Higher weekday time was marginally associated with higher AQ-Attention to Detail (*p* = 0.058), SPQ-Odd Speech, faster targeting times, and being male. Higher weekend time was marginally associated with AQ-Communication, SPQ-Odd speech, faster targeting times, and being male. Higher self-report spare time was associated with higher SPQ-Eccentric Behaviors, lower SPQ-Magical Thinking, faster targeting time, and being male. No statistically significant relationships were detected for reaction times and any of the time use variables. Results are summarized in Supplementary Table 13.

### Relationships of Reaction and Targeting Times With Video Game Usage

ANCOVAs were also conducted to analyze the relationships of neurophysiological indices (i.e., reaction and targeting times) and the five video game usage variables. Gender was significantly associated with all usage variables (Supplementary Table 14). In the case of self-reported frequency, males reported high baselines of video game use frequencies; a potential positive relationship between self-reported frequency and faster reaction time may have gone undetected due to a ceiling effect of general usage. In females, a strong positive relationship between self-reported video game use frequency and faster targeting times was detected.

### Relationships of Reaction and Targeting Times With Video Game Genre Preferences

By *t*-tests, faster reaction times were associated with preference for Action video games in both genders pooled together and marginally non-significantly in males (*p* = 0.07) (Supplementary Table 15). Faster reaction times were also associated with preference for RPG in both genders pooled together, in females, and in males. Strategy was also associated with faster reaction times in both genders pooled together and marginally non-significantly in females (*p* = 0.06). Construction was associated with faster reaction times with both genders pooled together and in females. Idle game use was associated with faster reaction time in both genders and in males only.

Preferences for Action and Idle game genres was associated with faster targeting time in both genders pooled together, and in females. Preferences for Role Playing Games (RPG), Strategy Games, and Construction Games were associated with faster targeting time in both genders, in females, and in males. Preferences for Social Simulation were associated with faster targeting time in females and males when they were analyzed separately.

ANCOVA was also conducted to analyze the separate relationships of reaction times, targeting times, to each of the 10 video game genres (Supplementary Table 16). Faster reaction times were associated with preference for Action, RPG, Strategy in in males. Faster reaction times showed a marginal association with preference for Construction (*p* = 0.066). Females reported greater preference for Puzzle and Social Simulation, but no significant relationships were found between reaction times and these two genres.

Faster targeting times were associated with preference for Action, RPG, Strategy, and Sports games in males. Faster targeting times were associated with preference for Construction and Idle, but no gender differences were detected. Females reported greater preference for Puzzle, but there were no significant differences in preference for Puzzle games and targeting times. Preference for Social Simulation were associated with faster targeting times in females. Results are presented in Supplementary Table 16.

By both types of analyses, preference for Action, RPG, Strategy were associated with faster reaction and targeting times in males. Faster reaction and targeting times were associated with preference for Construction in both genders together. Preference for Social Simulation was associated with faster targeting times in females. Females reported greater preference for Puzzle but Puzzle was not associated with either faster reaction or targeting times.

### Relationships of Reaction and Targeting Times With Video Game Motivations

By *t*-tests, faster reaction times were associated with the motivations Social Interaction, Skill Development, and Customization, in both genders pooled together and in males (Supplementary Table 17). Faster targeting times were associated with Social Interaction, Stress Relief, Skill Development, Adrenaline Rush, Escape, Fantasy, and Customization in both genders pooled together, males only, and females only.

By logistic regression analyses, Social Interaction was marginally non-significantly (*p* = 0.067) associated with faster reaction times in males (Supplementary Table 18). Skill Development was associated with faster reaction times in males. Males reported a greater preference for Adrenaline Rush, but no significant relationships were detected between Adrenaline Rush and faster reaction times. Customization was associated with faster reaction times. Social Interaction and Skill Development were associated with faster targeting time in males. Stress Relief was marginally non-significantly associated with faster targeting times (*p* = 0.051). Males reported greater preference for Adrenaline Rush, but no relationship were detected between Adrenaline Rush and targeting times. Fantasy and Customization were associated with faster targeting times.

By both types of analyses, Social Interaction and Skill Development were associated with faster reaction and targeting time in males. Customization was associated with both faster targeting and reaction time. Stress Relief and Fantasy were associated with faster targeting times.

## Discussion

The principal aims of this study were to investigate the relationships of autistic and schizotypal traits, gender, and select neurophysiological traits, with video game usage, preferences, and motivations. Table 2 summarizes the main results in the context of the predictions described in the Introduction. The most important findings are threefold. First, our results are largely consistent with prior findings that differences in patterns of video game usage mirror gender differences in childhood play, with some notable differences in females with higher autistic traits. Second, we have observed a male-typical pattern of video game usage, motivation, and genre preferences in females with higher autistic traits, which is in support of the Extreme Male Brain theory of autism (Baron-Cohen, 2002). Third, the results partially support Crespi and Badcock’s (2008) diametrical model of social brain disorders, in that some video game variables show opposite patterns between autism and positive schizotypy. We expand on each main result in turn, and then discuss the evolutionary psychological implications of virtual play on human social behaviors.

**TABLE 2:** Study findings in relation to the main predictions.

Predictions	Supported?	Findings
(1.1) Males will report greater video game usage than females.	Yes	Males reported higher video game usage across all time use variables.
(1.2) Males will prefer mechanistic-oriented video games (Action, Platformer, Strategy, Racing, Sports, RPG, Construction) while females will prefer Puzzle and Social Simulation video games.	Partially	Males reported greater preference for Action, Strategy, Sports, and RPG games. Females reported greater preference for Puzzle and Social Simulation games.
(1.3) Males will report greater motivation of Skill Development and Customization and females will report greater motivations of Social Interaction, Fantasy, and a way to Escape.	Partially	Males reported greater motivations of Social Interaction and Skill Development. Females with higher AQ reported greater motivations of Fantasy, Stress Relief, and Customization.
(2): Autistic traits will be associated with increased video game usage across all time use variables. SPQ-total and SPQ-CogPer will be associated with decreased video game usage across all time use variables.	Partially	Higher autistic traits were associated with greater video game usage in females. Higher SPQ-CogPer scores were associated with lower video game usage in both genders pooled together Higher autistic traits were not associated with greater video game usage in males.
(3): Autistic traits will be positively associated with preferences for Puzzle, Action, Platformer, Strategy, Racing, Sports, Idle, and Construction. SPQ-total and SPQ-CogPer will be positively associated with preferences for fantasy, RPG, and social simulation video games.	Partially	Females with higher AQ-Total preferred Construction and RPG games. Higher SPQ-CogPer scores and higher SPQ-Total scores were associated with greater preference for Puzzle games.
(4): Autistic traits will be positively associated with greater mechanistic motivations. SPQ-Total and SPQ-CogPer will be positively associated with mentalistic motivations.	Partially	Higher AQ-Total was associated with greater motivations of Stress Relief, Fantasy, and Customization. No associations were found for SPQ-Total or SPQ-CogPer.
(5): Autistic traits will be associated with faster reaction times and targeting times. SPQ-Total and SPQ-CogPer will be associated with slower reaction and targeting times.	Partially.	Higher SPQ-Magical Thinking was associated with slower targeting times.
(6): Increased total video game usage will be predicted by higher reaction and targeting times. In turn, slower reaction and targeting times will predict lower video game usage.	Yes	Faster reaction and targeting times were associated with greater video game usage.

### Gender Differences in Patterns of Video Game Usage

First, differences in video game usage patterns, genre preferences, and motivations are largely consistent with prior findings that males played more video games than females, preferred action type video games, and exhibited both faster reaction times (Greenberg et al., 2010) and enhanced targeting abilities (Watson and Kimura, 1991; Cook and Saucier, 2010). Our study has found that females reported greater gaming motives of Customization; by contrast, Hartmann and Klimmt (2006) found that females generally enjoy video games for their socially interactive aspects.

Why do males play video games more than females? One possibility may be that most video games are designed to simulate male-male individual or coalitional competition (Mendenhall et al., 2010), which are intrinsically rewarding and enjoyable to males (Chou and Tsai, 2007; Olson et al., 2007). For example, most first-person shooter (FPS) video games, such as Call of Duty, involve playing from the first-person view of a soldier. FPS gameplay mostly involves three main themes: (1) mastering the deployment of various weapons to destroy targets and enemies, (2) collaborating with other soldiers to accomplish missions, (3) rising through status hierarchies via achieving goals or collecting rewards and gear. In line with our results, previous studies on first-person shooter games have identified that they are mostly played by young males, who spend an average of 2.6 h a day on gaming, and report social interaction, competition, and challenge as their main motives for gaming (Jansz and Tanis, 2007). Likewise, MMORPGs, such as World of Warcraft (reported to consist of 85% of male user-base (Ducheneaut et al., 2006) are designed to promote inter-factional warfare, the collection of rare and conspicuous avatar equipment (i.e., status signaling) and collaborative practices that simulate conditions of hunting (Mendenhall et al., 2010). Altogether, the greater representation of male video game players appears to reflect the male-typical competitive environments of most video game genres, which seek to recreate or simulate settings of cooperative hunting and warfare.

The findings reported here are also consistent with prior research indicating that females prefer classic puzzle or board games (Greenberg et al., 2010) and life simulation games (Nepomuceno et al., 2010; Yee, 2017), the latter of which tends to be more exploratory in nature and with a greater emphasis on social relationships than competition. Interestingly, although females generally reported a preference for Social Simulation games, they did not cite Social Interaction as a motivation for using video games. Contrary to the hypothesis posited here, females reported Customization as their primary motivation. Two possibilities may be at play here. First, given that females with higher autistic traits play more video games (as discussed in more detail below), the female pattern of video game motivation may reflect the higher mechanistic (i.e., non-social) preferences of the autistic phenotype. That is, given that autistic traits have been associated with greater preference for building activities and solitary play (Holmes and Willoughby, 2005), the identification of Customization, rather than Social Interaction, as the main gaming motive, may reflect the systemizing bias of autism. Secondly, given that females are more likely to be casual video game players than males, and casual video game players tend to rank higher on extraversion (Potard et al., 2019), females (at least ones with lower AQ scores) may have other social outlets for meeting interpersonal needs outside video games, and thus be less likely to identify Social Motivation as a main motive for video game usage.

Females with higher levels of autistic traits also preferred male-typical video game preferences, including RPG and Construction, relative to general female-typical genre preferences such as Puzzle and Social Simulation. A preference for Construction type games was not detected among males, although previous studies have documented male preferences for constructive toys (Blakemore and Centers, 2005). As for males with higher AQ, females with higher AQ also identified the mechanistic preference Customization as one of their main gaming motives.

### Extreme Male Brain Theory of Autism and Patterns of Video Game Usage

Our second hypothesis predicted that autistic traits will be associated with increased video game usage. Interestingly, higher autistic traits were associated with greater video game usage, but only in females and not in males. The pattern of females with higher total autism scores playing video games more frequently is of notable interest, as it fits with Baron-Cohen’s (2002) extreme male brain theory of autism, and partially with the predictions from Crespi and Badcock (2008) and Wendt et al. (2019). Thus, females with higher AQ-total scores were “more male” in their pattern of higher video game usage. Analysis of the AQ subscales revealed that that decreased social skills, higher attention to detail, and lower social imagination showed evidence of being related to greater video game usage in females with higher autistic traits. Taken together, the cluster of autistic traits outlined above suggest that higher video game usage in females may be attributable to a more systemizing cognitive style (Baron-Cohen, 2010). Indeed, the association between higher autistic traits in females and greater motives of Customization and preference for Construction-type games is consistent with such view. For example, one popular Construction game, Minecraft, focuses predominately on the mechanistic drive of building objects. As the virtual environment is literally made of pixelated blocks, gameplay is centered around manipulating objects in systematic ways to construct buildings, mazes, and even entire cities. Paralleling play behaviors in children with autism, building activities in Minecraft are usually solitary, detail-oriented, and reality-based. For example, “making” a book in Minecraft, as with real life, requires arranging virtual pieces of paper with virtual leather in a particular order for the object to be realized. Given that people with autism are disproportionately represented in the engineering field (Baron-Cohen et al., 1997; Baron-Cohen, 1998) and children with autism tend to focus on functional play (i.e., play with an object for its function) and constructive play (i.e., building sand castles), preference for parallel (i.e., playing alongside others but not together) or solitary play (Holmes and Willoughby, 2005), the patterns of video game usage described above appear to be an extension of autistic play behaviors to in a virtual, computerized medium.

Females with higher total autism scores also reported greater gaming motivations of Stress Relief, Fantasy, and Customization; the first two motivations may be categorized under social fulfillment via virtual play, and the latter may represent an autism-related preference for rule-based, solitary mechanistic interest such as building objects (e.g., Minecraft). Taken together, these patterns suggest that higher autistic traits may be related to greater video game usage via a decreased capacity for, or interest in, social and pretend play. In other words, because most video games environments involve pre-programmed characters, narratives, and behaviors, they may appeal to individuals with higher autistic traits who prefer predictable environments that can be navigated with pre-defined action patterns. In this light, video games may also function specifically for their self-contained immersive experiences that provide the illusion of sharing a social world without partaking in one. Furthermore, the relative anonymity and use of virtual avatars in video games offer an additional layer of interpersonal distance that can be conceptualized as the virtual extensions of parallel play behaviors commonly observed in children with autism (Holmes and Willoughby, 2005). Users are thus playing “together” but remain alone. Moreover, the pattern of autistic game play pattern described here can be differentiated from general play differences in two important ways: (1) although females with higher autistic traits report male-typical preferences such as RPG and Customization, they do not share the other male-typical preferences that were more socially collaborative in nature, such as Sports, or Action and Strategy genres that involve extensive teamwork (ex: military group-collaborations in Call of Duty); and (2) the identification of Fantasy as a motivation was not associated with the concurrent motivation of Social Interaction, which suggest that solitary play was the common denominator described here.

Why was there no evidence of a positive correlation of AQ score with video game use among males? One possibility is a “ceiling effect,” whereby males would exhibit too little variation in video game use scores for a positive relationship to be detected. However, males do exhibit comparable levels of variability (as measured by standard deviations) to females across the various measures of video game usage, and positive correlations restricted to females were found for multiple video game use frequency measures, which appears to render major influences from ceiling effects relatively unlikely. Alternatively, because most video games are designed with male-typical preferences such as competition (Jansz and Tanis, 2007) and physical violence (Jansz, 2005), and females are more likely to prefer non-violent entertainment ventures (Slater, 2003) and may be less competitive than males (Deaner, 2013), video game usage among females with higher total AQ scores may be more pronounced, compared to males, with high-AQ females demonstrating more male-typical cognitive phenotypes relative to the general female population.

### The Diametrical Model of Social Brain Disorders and Patterns of Video Game Usage

Our second hypothesis also predicted that patterns of video game usage would vary in opposite patterns along the autism-schizotypal axis, with autistic traits associated with greater video game usage and positive schizotypal traits (i.e., SPQ-Total and SPQ-CogPer) with lower video game usage.

Evidence of negative relationships of SPQ-CogPer and SPQ-Magical Thinking with video game usage was detected from several analyses. These findings partially support predictions of the Crespi and Badcock (2008) model, in that autistic and positive schizotypy traits predicted greater and lower video game usage, respectively (though with gender-limited effects for autism that were not predicted by the model). Given that SPQ-Magical Thinking was also associated with slower targeting times, lower video game usage of individuals with high levels of this psychological trait may stem, in part, from general hand-eye motor speed and coordination difficulties from executing particular action patterns necessary for effective console/controller use. Indeed, a positive relationship was detected between SPQ-CogPer and Puzzle games, but Puzzle was not associated with faster reaction or targeting times. Furthermore, although both Puzzle and Social Simulation games are generally designed to be played at leisure, the latter was associated with faster targeting times but not the former, further providing support that variation in neurophysiological indices may be contributing to the overall lower video game usage.

Why would individuals higher in positive schizotypal traits play video games less frequently? As outlined above, most video games appear to offer immersive but ultimately solitary play experiences that may not fulfill the mentalistic interests of many schizotypal individuals. Historically, most video games were developed as single-player ventures; lack of historical internet access meant that most games were developed to be enjoyed with only a limited number of players. For examples, the popularity of PC-based games in the 1990s meant that most games were designed to be self-contained, with clearly defined objectives, and catering to single players. In this light, given that the Crespi and Badcock (2008) model predicts that autistic and schizotypal traits would vary in opposite directions in socio-cognitive development—what would be the theoretical equivalent of autism and videogames for positive schizotypy?

An opposite medium (as opposed to different games *per se*) would theoretically contain the following characteristics of high positive schizotypy: (1) rely on a short and fractured attention span on a wide variety of inter-changeable topics instead of one highly concentrated focus on a restricted set of clearly defined objectives, (2) verbally based, (3) high social connectivity, (4) virtual elements of high social monitoring in terms of detectable social feedback signals (i.e., likes, followers) that mimic in-person cues of joint attention and/or eye contact. In short, the logical equivalent of autism-video games appears to be schizotypy-Twitter or TikTok. Although we did not explicitly test for the relationship between schizotypy and social media platforms here, the association between positive schizotypy and Puzzle games suggest that such preferences could theoretically be extrapolated to social media platforms. Like most Puzzle games, most social media applications are designed to be used leisurely, with less cognitively taxing actions, and with no penalties if the user has less than accurate targeting movements.

Several explanations have been proposed for gender differences in reaction and targeting time. First, given that playing action video games can improve reaction time (Chandra et al., 2016), the gender differences may stem from the strong male preference toward action-oriented video games that require fast decision-making and projectile analysis in a simulated 3-D space. Our data support the training effects of video game usage in that in both genders, reaction and targeting times were positively associated with greater video game usage. Second, males also reported greater motivation for using video games for Skill Development, another factor that could contribute to the development of faster reaction and targeting time due to greater intrinsic motivation for improvement. Alternatively, the gender differences in reaction and targeting time may stem from evolved physiology in sexual divisions of labor; that is, males may have evolved faster reaction and targeting times than females due to greater neurophysiological demands of hunting and competition that have characterized the human hunter-gatherer past (Cherney and Poss, 2008). Finally, given that SPQ-Magical Thinking was associated with slower targeting times and that females scored higher on SPQ-Magical Thinking than did males, the gender differences in targeting time may be linked with general gender differences in levels of this schizotypal trait. Indeed, the association of higher Magical Thinking with slower targeting time is consistent with previous literature showing that higher positive schizotypy is associated with higher prediction error rates on hand-eye coordination tasks (Asai et al., 2008) and increased dyscontrol in fine motor movements (Roché et al., 2015). These findings may, more generally, reflect the higher levels of sensory and sensorimotor deficits found among individuals on the schizophrenia spectrum (Javitt and Freedman, 2015).

Second, in accordance with the Crespi and Badcock (2008) model, autistic traits were generally associated with mechanistic genre preferences such as Construction and RPG. However, contrary to the predictions made here, higher SPQ-CogPer scores and higher SPQ-Total scores were associated with greater preference for Puzzle games. Theoretically, puzzle games could be considered a mechanistic-typical genre as most traditional puzzles involve deducing or reverse-engineering a set of given patterns to arrive at the correct solution. What would explain this finding? One possibility is that most virtualized puzzle games do not involve the same depth or rigor of algorithmic thinking as it would in traditional puzzle games. For example, one of the most popular app-based puzzle games, Candy Crush, involves detecting a pattern of three or more identical shapes and swiping them away with ones’ fingers; gameplay is largely easy and simple pattern-detection in a 2-D space. In contrast, a traditional puzzle game might be solving the Magic Cube, a task that involves simultaneous operations of 3-D mental rotation, mathematical operations, and mental simulation of object transformations in horizontal/vertical space. Thus, although both physical and virtual “puzzle” games may appear similar, the constraints of representing games in a virtual 2-D space may remove or imbue it with different functions that may render it considerably different from its original counterpart.

The association of positive schizotypal traits with Puzzle type games is consistent with previous findings that patients with schizophrenia rated puzzle-type games as one of their top three preferred game genres (i.e., board/card games) (Choi et al., 2020). Surprisingly, contrary to the predictions made here, positive schizotypal traits were not related to more socially oriented or fantasy-based genres such as RPG or Social Simulation. Two possible explanations may help to account for this result. First, most popular role-playing video game games, such as World of Warcraft, are cognitively demanding and involve juggling of multiple simultaneous tasks (i.e., switching between weapons, building and maintaining multiple coalitions, tracking and shooting at multiple targets), which may be challenging given that schizotypy has been associated with attentional impairments under situations of high perceptual load (Stotesbury et al., 2018). In comparison, Puzzle-type games, such as Candy Crush, tend to less time-intensive, more casual, and designed to be played at leisure with no penalties if the user stops at random intervals. Second, the most popular current Social Simulation franchise, such as Animal Crossing, involve considerable components of customization (e.g., building and furnishing houses, gathering, and collecting items) which may be more mechanistically inclined and less appealing to individuals with higher schizotypal traits.

Lastly, patterns of video game motivations described here partially support the Crespi and Badcock (2008) model that autistic traits predict mechanistic motivations for playing video games (i.e., Customization). Interestingly, AQ-total was also associated with Fantasy, but not Social Interaction, motivations. The pattern described here is suggestive that video games provide an evolutionarily novel play environment where fantasy play can be decoupled from social interactions. In other words, although autistic traits were associated with motivations for utilizing video games for Stress Relief and Fantasy motives, it was not associated with motivations for socially interacting with others, which suggests that although, on the surface, most role-playing games involve multiplayer modes and opportunities for social interactions, individuals with autistic traits seem to partake in them for the immersive experience but not the social aspect. Thus, utilizing video games for fantasy play appears to differ from real-life fantasy play in the important aspect that the imaginative component could be dissociated from its corresponding social component, which are intricately linked together most real-life, in-person childhood pretend play.

### Limitations

Our study is constrained by several limitations. First, the data were collected during a school semester; thus, general video game usage patterns may have been under-reported due to greater time demands of the typical school semester. Future studies may account for potential differences in video game usage patterns due to classwork demands by incorporating a non-typical school semester in its time span of data collection for comparison. Second, given that we primarily recruited through a psychology student pool as well as university email lists, most of participants may have come from the undergraduate population; thus, the findings cannot be extrapolated to clinical populations, children, the elderly, or professional gamers who may demonstrate psychological traits atypical of our sample. Third, given that autistic and schizotypal traits were assessed via self-reports, results may be limited in that higher autistic and schizotypal traits may, paradoxically, be associated with lower cognitive insights and thus under-report of traits. Fourth, given the heterogeneous nature of most video games (ex: Legend of Zelda is classified as Action on most commercial video game websites, but it also involves considerable role-playing and puzzle-solving), findings from this study may be limited due to blending of mechanistic vs. mentalistic gameplay that characterize some video games. Future research could address such limitations by adding an open-feedback answer item as to better disentangle nuances in users’ motivations for gaming and gaming preferences. Finally, given that we administered reaction time and targeting time tasks online, variability in reaction and targeting time data may be confounded by participants’ choice of electronic device to complete the tasks (ex: computer with mouse, laptop, iPad or another hand-held device with touchpad). Although the computer tasks were designed to accommodate a wide variety of electronic devices, variability in reaction time and targeting time data may be influenced by participants’ use of a mouse vs. touchpad, which we did not control for. Some studies have shown that individuals were slower and tend to make more errors when using a touchpad than mouse to make pointing actions (Hertzum and Hornbæk, 2010), and that using the mouse was faster than touchpads or MultiTouch devices in cursor positioning (Shanis and Hedge, 2003). Given that our study was conducted remotely and thus we could not verify what sort of hardware participants used, future research could collect data on the type of device used to control for any potential confounds.

Since the COVID pandemic began, more and more recreational platforms have been moving to the virtual world. What will become of iGen 2030? The patterns described above suggest that the replacement of in-person play with virtual media may have important implications for the evolution of imaginative culture. First, the patterns in autistic play behaviors and video game usage described in this study suggest that video games may be a proverbial “new and reinvented” version of fantasy play. That is, unlike a traditional fantasy game where people must spontaneously imagine a shared mental space and participate accordingly, video games offer a pre-programmed imaginative space where anyone can join without having to spontaneously imagine entities into existence. Moreover, for the first time in human history, people are no longer bounded by the temporal-spatial limitations of other players to start and maintain a game. Almost everyone now can play a video game together—and alone.

Finally, although we have found some evidence of decreased video game usage in association with positive schizotypy, we did not observe statistically significant patterns for greater mentalistic preferences in positive schizotypy as we did for mechanism and autism. Why would this be? One possibility is that video games only represent one end of the spectrum of virtual play behaviors that has now substituted for much of real-life play. The patterns of video game usage, preferences, and motivations described above suggest that imaginative and social aspects could be dissociated in video games; individuals with higher autistic traits appear to partake more in the former in lieu of the latter. In this light, as Crespi and Badcock (2008) postulated autism and psychosis as two ends of a continuum of social cognitive development, so there may exist a similar virtual gradient of play behaviors that may be expressed in two opposites but related media. What could be on the other end of the spectrum? Given that most social media platforms are highly socially oriented, verbally based, and performative, future studies could investigate potential relationships of social media use and positive schizotypy given the former’s mentalistic bias.

## Data Availability Statement

The raw data supporting the conclusions of this article will be made available by the authors, without undue reservation.

## Ethics Statement

The studies involving human participants were reviewed and approved by the Simon Fraser University Department of Research Ethics. The participants provided their written informed consent to participate in this study.

## Author Contributions

All authors listed have made a substantial, direct, and intellectual contribution to the work, and approved it for publication.

## Conflict of Interest

The authors declare that the research was conducted in the absence of any commercial or financial relationships that could be construed as a potential conflict of interest.

## Publisher’s Note

All claims expressed in this article are solely those of the authors and do not necessarily represent those of their affiliated organizations, or those of the publisher, the editors and the reviewers. Any product that may be evaluated in this article, or claim that may be made by its manufacturer, is not guaranteed or endorsed by the publisher.

## Footnotes

1
https://www.psytoolkit.org/

2
www.surveymonkey.com
