# Supplementary figures and images for: Why iPlay: The Relationships of Autistic and Schizotypal Traits With Patterns of Video Game Use
Source: Front Psychol. 2022 Feb 23;13:767446. doi: 10.3389/fpsyg.2022.767446 (PMC8905237; doi:10.3389/fpsyg.2022.767446)

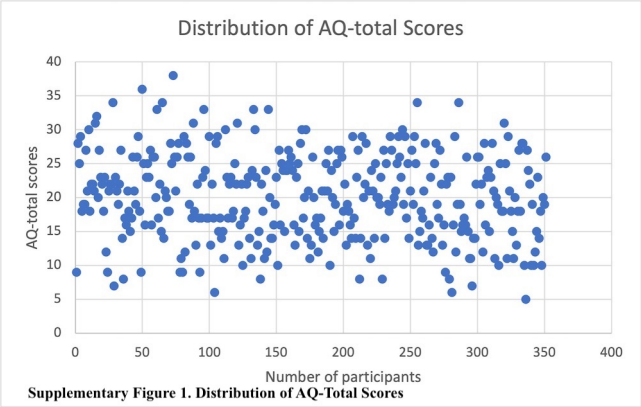

Supplement: Supplementary file 1 [file Image_1.pdf]

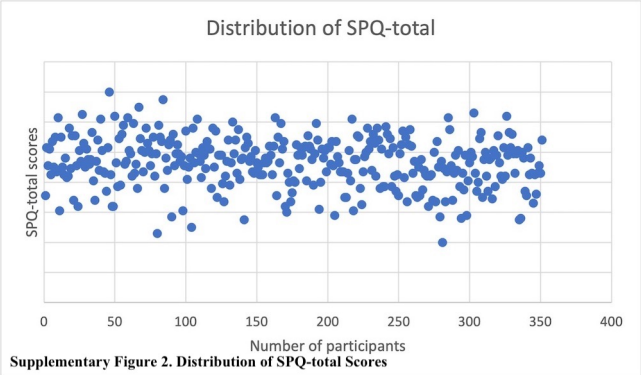

Supplement: Supplementary file 2 [file Image_2.pdf]
