# Supplementary material for: Why iPlay: The Relationships of Autistic and Schizotypal Traits With Patterns of Video Game Use
Source: Front Psychol. 2022 Feb 23;13:767446. doi: 10.3389/fpsyg.2022.767446 (PMC8905237; doi:10.3389/fpsyg.2022.767446)
Supplement: Supplementary file 3 [file Data_Sheet_1.docx]

Supplementary Material

**Supplementary Table 1**. Welch's t-tests on Sex Differences in AQ and SPQ, Video Game Usage, and Reaction Times and Targeting Times

| **Variables** | **Scores**  **Mean ± SD (N)** | | | **t (df)** | **p value** |
| --- | --- | --- | --- | --- | --- |
| **AQ** | | | | | |
| Social | F | 3.50 | ± 2.49 (207) | **1.91 (313)** | 0.06 |
|  | M | 2.95 | ± 2.43 (144) |  |  |
| AQ- Switch | F | 5.73 | ± 1.91 (207) | 1.05 (305) | 0.30 |
|  | M | 5.51 | ± 1.94 (144) |  |  |
| AQ- Detail | F | 5.34 | ± 2.07 (144) | 1.08 (327) | 0.28 |
|  | M | 5.59 | ± 2.31 (207) |  |  |
| AQ- Comm | F | 3.22 | ± 2.19 (207) | 0.12 (307) | 0.91 |
|  | M | 3.19 | ± 2.20 (144) |  |  |
| AQ- Imag | F | 2.50 | ± 1.54 (207) | 0.05 (296) | 0.96 |
|  | M | 2.49 | ± 1.63 (144) |  |  |
| AQ: Total | F | 20.51 | ± 6.41 (207) | 1.47 (309) | 0.14 |
|  | M | 19.49 | ± 6.38 (144) |  |  |
| **SPQ** | | | | | |
| SPQ- Ideas | F | 18.91 | ± 4.50 (207) | **2.62 (294)** | **0.009** |
|  | M | 17.58 | ± 4.81 (144) |  |  |
| SPQ- Constrict | F | 16.88 | ± 5.12 (207) | -1.30 (319) | 0.20 |
|  | M | 17.58 | ± 4.83 (144) |  |  |
| SPQ- Eccentric | F | 10.96 | ± 3.93 (207) | -0.94 (326) | 0.35 |
|  | M | 11.34 | ± 3.56 (144) |  |  |
| SPQ- Anxiety | F | 13.87 | ± 3.96 (207) | **3.61 (302)** | **3.57E-04** |
|  | M | 12.29 | ± 4.08 (144) |  |  |
| SPQ- Magic | F | 9.45 | ± 3.73 (207) | **4.88 (315)** | **1.69E-06** |
|  | M | 7.52 | ± 3.58 (144) |  |  |
| SPQ- Speech | F | 14.09 | ± 3.10 (207) | **2.33 (300)** | **0.02** |
|  | M | 13.29 | ± 3.23 (144) |  |  |
| SPQ- Percep | F | 10.81 | ± 3.07 (207) | 1.62 (303) | 0.11 |
|  | M | 10.26 | ± 3.14 (144) |  |  |
| SPQ- Interpersonal | F | 30.74 | ± 7.54 (207) | 1.09 (312) | 0.28 |
|  | M | 29.86 | ± 7.40 (144) |  |  |
| Disorganized | F | 94.97 | ± 16.20 (207) | **2.87 (306)** | **0.004** |
|  | M | 89.85 | ± 16.58 (144) |  |  |
| Cog-SPQ- Percep | F | 39.17 | ± 8.36 (207) | **4.04 (296)** | **6.72E-05** |
|  | M | 35.37 | ± 8.89 (144) |  |  |
| SPQ: Total | F | 94.97 | ± 16.20 (207) | **2.87 (303)** | **0.004** |
|  | M | 89.85 | ± 16.58 (144) |  |  |
| **Video Game Usage** | | | | | |
| Weekday Time (h) | F | 0.83 | ± 1.29 (207) | **-7.96 (252)** | **5.89E-14** |
|  | M | 2.17 | ± 1.71 (144) |  |  |
| Weekend Time (h) | F | 1.56 | ± 1.90 (207) | **-9.10 (260)** | **2.40E-17** |
|  | M | 3.75 | ± 2.41 (144) |  |  |
| Self Report Frequency | F | 3.66 | ± 2.16 (207) | **11.79 (349)** | **2.03E-27** |
|  | M | 5.93 | ± 1.45 (144) |  |  |
| Self Report Usage | F | 3.44 | ± 1.44 (207) | **6.85 (329)** | **3.645E-11** |
|  | M | 2.44 | ± 1.28 (144) |  |  |
| Self Report Spare Time | F | 1.84 | ± 1.02 (207) | **-8.90 (282)** | **7.13E-17** |
|  | M | 2.90 | ± 1.16 (144) |  |  |
| **Neurophysiological Indices** | | | | | |
| Reaction time (ms) | F | 286.28 | ± 33.95 (204) | **-3.95 (302)** | **9.84E-05** |
|  | M | 271.44 | ± 34.93 (144) |  |  |
| Target Time (ms) | F | -163.20 | ± 100.22 (195) | **-5.89 (280)** | **1.08E-08** |
|  | M | -93.53 | ± 111.07 (140) |  |  |

**Supplementary Table 2**. Chi-Square Tests for Sex Differences in Video Game Genre Preferences and Motivations

| Genre |  | | N | χ^2^ | p |
| --- | --- | --- | --- | --- | --- |
| Puzzle | F | Yes | 107 | 16.54 | 0.00005 |
|  |  | No | 100 |  |  |
|  | M | Yes | 43 |  |  |
|  |  | No | 101 |  |  |
| Action | F | Yes | 61 | 77.04 | < 0.00001 |
|  |  | No | 146 |  |  |
|  | M | Yes | 111 |  |  |
|  |  | No | 33 |  |  |
| Platformer | F | Yes | 65 | 0.76 | 0.39 |
|  |  | No | 142 |  |  |
|  | M | Yes | 39 |  |  |
|  |  | No | 105 |  |  |
| RPG | F | Yes | 44 | 34.55 | < 0.00001 |
|  |  | No | 163 |  |  |
|  | M | Yes | 74 |  |  |
|  |  | No | 70 |  |  |
| Strategy | F | Yes | 28 | 48.53 | < 0.00001 |
|  |  | No | 179 |  |  |
|  | M | Yes | 68 |  |  |
|  |  | No | 76 |  |  |
| Sports | F | Yes | 29 | 14.11 | 0.0002 |
|  |  | No | 178 |  |  |
|  | F | Yes | 44 |  |  |
|  |  | No | 100 |  |  |
| Racing | F | Yes | 44 | 0.05 | 0.83 |
|  |  | No | 163 |  |  |
|  | M | Yes | 32 |  |  |
|  |  | No | 112 |  |  |
| Construction | F | Yes | 65 | 2.51 | 0.11 |
|  |  | No | 142 |  |  |
|  | M | Yes | 57 |  |  |
|  |  | No | 87 |  |  |
| Social Simulation | F | Yes | 76 | 22.27 | < 0.00001 |
|  |  | No | 131 |  |  |
|  | M | Yes | 20 |  |  |
|  |  | No | 124 |  |  |
| Idle | F | Yes | 20 | 0.05 | 0.82 |
|  |  | No | 187 |  |  |
|  | M | Yes | 15 |  |  |
|  |  | No | 129 |  |  |
| Motivations |  |  | **N** | **χ^2^** | **p** |
| Social interaction | F | Yes | 61 | 19.39 | 0.00001 |
|  |  | No | 146 |  |  |
|  | M | Yes | 76 |  |  |
|  |  | No | 68 |  |  |
| Stress relief | F | Yes | 131 | 3.07 | 0.08 |
|  |  | No | 76 |  |  |
|  | M | Yes | 104 |  |  |
|  |  | No | 40 |  |  |
| Skill Development | F | Yes | 33 | 16.59 | 0.00005 |
|  |  | No | 174 |  |  |
|  | M | Yes | 50 |  |  |
|  |  | No | 94 |  |  |
| Adrenaline Rush | F | Yes | 46 | 13.28 | 0.0003 |
|  |  | No | 161 |  |  |
|  | M | Yes | 58 |  |  |
|  |  | No | 86 |  |  |
| Escape | F | Yes | 116 | 1.80 | 0.18 |
|  |  | No | 91 |  |  |
|  | M | Yes | 91 |  |  |
|  |  | No | 53 |  |  |
| Fantasy | F | Yes | 49 | 7.83 | 0.005 |
|  |  | No | 158 |  |  |
|  | M | Yes | 54 |  |  |
|  |  | No | 90 |  |  |
| Customization | F | Yes | 58 | 4.03 | 0.04 |
|  |  | No | 149 |  |  |
|  | M | Yes | 55 |  |  |
|  |  | No | 89 |  |  |

**Supplementary Table 3**. ANCOVAs of Total AQ, Total SPQ, and SPQ Cog-Per scores in relation to Video Game Usage, with Sex as Interaction Term

| **Weekday time (h)** | | | | | | |
| --- | --- | --- | --- | --- | --- | --- |
|  | **t trait** | **p trait** | **t sex** | **p sex** | **t interact** | **p interact** |
| AQ- total | 1.65 | 0.101 | 3.80 | 0.00017 | -1.28 | 0.201 |
| SPQ- Cog-Per | -0.63 | 0.530 | 1.94 | 0.053 | -0.10 | 0.923 |
| SPQ- total | -0.30 | 0.767 | 0.92 | 0.361 | 0.56 | 0.578 |
| **Weekend time (h)** | | | | | | |
|  | **t trait** | **p trait** | **t sex** | **p sex** | **t interact** | **p interact** |
| AQ- total | **2.73** | **0.007** | **4.21** | **0.00003** | **-1.29** | **0.197** |
| SPQ- Cog-Per | -0.81 | 0.420 | 1.95 | 0.052 | 0.16 | 0.874 |
| SPQ- total | 0.69 | 0.488 | 1.56 | 0.119 | 0.13 | 0.900 |
| **Self Report Frequency** | | | | | | |
|  | **t trait** | **p trait** | **t sex** | **p sex** | **t interact** | **p interact** |
| AQ- total | **2.81** | **0.005** | **5.34** | **0.0000002** | **-2.01** | **0.045** |
| SPQ- Cog-Per | **-1.93** | **0.054** | **1.80** | 0.072 | 0.62 | 0.536 |
| SPQ- total | -0.15 | 0.883 | 1.95 | 0.052 | -0.04 | 0.971 |
| **Self Report Usage** | | | | | | |
|  | **t trait** | **p trait** | **t sex** | **p sex** | **t interact** | **p interact** |
| AQ- total | **-3.07** | **0.002** | **-4.57** | **6.72E-06** | **2.58** | **0.010** |
| SPQ- Cog-Per | -0.59 | 0.553 | -2.06 | 0.040 | 0.54 | 0.590 |
| SPQ- total | -1.57 | 0.117 | -1.45 | 0.147 | 0.23 | 0.819 |
| **Self report spare time** | | | | | | |
|  | **t trait** | **p trait** | **t sex** | **p sex** | **t interact** | **p interact** |
| AQ- total | 1.45 | 0.147 | 3.19 | 0.002 | -0.40 | 0.693 |
| SPQ- Cog-Per | **-2.30** | **0.022** | 0.82 | 0.414 | 1.19 | 0.236 |
| SPQ- total | -0.45 | 0.655 | 0.31 | 0.754 | 1.32 | 0.189 |

**Supplementary Table 4.** Pearson Correlations of AQ and SPQ subscale scores with Measures of Video Game Usage

| Video Game Usage | | | | | |
| --- | --- | --- | --- | --- | --- |
|  | **Weekday time (h)** | **Weekend time (h)** | **Self report frequency** | **Self report usage** | **Self report spare time** |
| Females (N = 207) |  |  |  |  |  |
| AQ: Social | 0.11 | **0.18**** | **0.18**** | **-0.17*** | 0.12 |
| AQ: Switch | -0.04 | 0.04 | 0.06 | -0.06 | 0.02 |
| AQ: Detail | **0.16*** | 0.12 | 0.06 | -0.04 | 0.06 |
| AQ: Comm | 0.04 | 0.12 | 0.07 | -0.12 | 0.06 |
| AQ: Imag | 0.11 | **0.17*** | **0.15*** | -0.10 | 0.07 |
| SPQ: Ideas | -0.03 | -0.01 | -0.09 | -0.04 | -0.11 |
| SPQ: Constrict | -0.12 | -0.02 | 0.00 | -0.07 | -0.04 |
| SPQ: Eccentric | 0.05 | 0.13 | 0.06 | -0.12 | 0.13 |
| SPQ: Anxiety | 0.08 | **0.17*** | 0.12 | -0.11 | 0.11 |
| SPQ: Magic | -0.03 | -0.11 | -0.11 | -0.02 | **-0.15*** |
| SPQ: Speech | 0.04 | 0.10 | 0.04 | -0.03 | 0.04 |
| SPQ: Percep | -0.06 | -0.03 | -0.05 | -0.03 | -0.13 |
| SPQ: Interpersonal | -0.04 | 0.08 | 0.07 | -0.11 | 0.03 |
| SPQ: Disorganized | 0.07 | **0.14*** | 0.06 | -0.10 | 0.11 |
| Males (N = 144) |  |  |  |  |  |
| AQ: Social | -0.09 | 0.06 | -0.001 | 0.08 | 0.07 |
| AQ: Switch | -0.01 | -0.01 | -0.02 | 0.12 | -0.05 |
| AQ: Detail | 0.06 | 0.02 | -0.01 | -0.06 | 0.05 |
| AQ: Comm | 0.05 | 0.14 | 0.04 | -0.09 | 0.18* |
| AQ: Imag | -0.08 | -0.14 | -0.06 | 0.15 | -0.13 |
| SPQ: Ideas | 0.06 | 0.06 | -0.05 | -0.06 | 0.06 |
| SPQ: Constrict | -0.03 | 0.00 | 0.03 | -0.09 | 0.08 |
| SPQ: Eccentric | 0.10 | 0.13 | 0.14 | **-0.20*** | **0.18*** |
| SPQ: Anxiety | 0.08 | 0.07 | 0.03 | -0.02 | 0.12 |
| SPQ: Magic | -0.11 | -0.08 | -0.14 | **0.17*** | **-0.17*** |
| SPQ: Speech | 0.14 | 0.15 | -0.05 | -0.15 | 0.15 |
| SPQ: Percep | -0.11 | -0.10 | -0.03 | -0.05 | -0.001 |
| SPQ: Interpersonal | 0.03 | 0.04 | 0.01 | -0.07 | 0.12 |
| SPQ: Disorganized | 0.14 | 0.16 | 0.08 | **-0.20*** | **0.19*** |
| Both Sexes(N=351) |  |  |  |  |  |
| AQ: Social | -0.03 | 0.07 | 0.05 | -0.03 | 0.04 |
| AQ: Switch | 0.05 | -0.01 | -0.02 | 0.03 | -0.04 |
| AQ: Comm | 0.04 | 0.11 | 0.04 | -0.09 | 0.09 |
| AQ: Attention to Detail | 0.03 | 0.02 | 0.07 | 0.003 | -0.01 |
| SPQ: Ideas | -0.05 | -0.04 | **-0.13**** | 0.01 | -0.08 |
| SPQ: Constrict | -0.04 | 0.02 | 0.03 | -0.10 | 0.05 |
| SPQ: Eccentric | 0.08 | **0.14*** | 0.09 | **-0.15**** | **0.16**** |
| SPQ: Anxiety | -0.01 | 0.02 | -0.03 | -0.001 | 0.02 |
| SPQ: Magic | **-0.16**** | **-0.20***** | **-0.23***** | **0.13**** | **-0.25***** |
| SPQ: Speech | 0.03 | 0.05 | -0.05 | -0.03 | 0.02 |
| SPQ: Percep | **-0.11*** | -0.09 | -0.81 | -0.01 | -0.10 |
| SPQ: Interpersonal | -0.03 | 0.03 | 0.01 | -0.06 | 0.04 |
| SPQ: Disorganized | 0.05 | **0.12*** | 0.03 | **-0.11*** | **0.11*** |

* < denotes significance at <0.05

** < denotes significance at < 0.01

*** denotes significance at < 0.001

**Supplementary Table 5**. ANCOVAs of AQ and SPQ subscales in relation to Video

Game Usage, with Sex as Covariate

| **Weekday time (h)** | | | | |
| --- | --- | --- | --- | --- |
|  | **t trait** | **p trait** | **t sex** | **p sex** |
| AQ- Social | 0.26 | 0.80 | 8.33 | 1.82663E-15 |
| AQ- Switch | -0.47 | 0.64 | 8.31 | 2.12419E-15 |
| AQ- Detail | 2.14 | 0.03 | 8.51 | 5.16871E-16 |
| AQ- Comm | 0.88 | 0.38 | 8.36 | 1.47431E-15 |
| AQ- Imag | 0.30 | 0.76 | 8.35 | 1.61269E-15 |
| SPQ- Ideas | 0.25 | 0.80 | 8.30 | 2.27628E-15 |
| SPQ- Constrict | -1.38 | 0.17 | 8.45 | 8.18346E-16 |
| SPQ- Eccentric | 1.33 | 0.19 | 8.30 | 2.4011E-15 |
| SPQ- Anxiety | 1.50 | 0.13 | 8.51 | 5.24111E-16 |
| SPQ- Magic | -1.22 | 0.22 | 7.79 | 7.57637E-14 |
| SPQ- Speech | 1.65 | 0.10 | 8.52 | 4.79066E-16 |
| SPQ- Percep | -1.50 | 0.13 | 8.22 | 4.21926E-15 |
|  | -0.12 | 0.91 | 8.33 | 1.89144E-15 |
| **Weekend time (h)** | | | | |
|  | **t trait** | **p trait** | **t sex** | **p sex** |
| AQ- Social | 2.36 | 0.02 | 9.74 | 5.47377E-20 |
| AQ- Switch | 0.33 | 0.74 | 9.48 | 4.05245E-19 |
| AQ- Detail | 1.47 | 0.14 | 9.57 | 2.00166E-19 |
| AQ- Comm | 2.48 | 0.01 | 9.57 | 1.98204E-19 |
| AQ- Imag | 0.39 | 0.70 | 9.48 | 4.11111E-19 |
| SPQ- Ideas | 0.48 | 0.63 | 9.45 | 5.00629E-19 |
| SPQ- Constrict | -0.15 | 0.88 | 9.46 | 4.60038E-19 |
| SPQ- Eccentric | 2.42 | 0.02 | 9.42 | 6.23987E-19 |
| SPQ- Anxiety | 2.32 | 0.02 | 9.82 | 3.0562E-20 |
| SPQ- Magic | -1.85 | 0.07 | 8.75 | 9.09994E-17 |
| SPQ- Speech | 2.29 | 0.02 | 9.76 | 4.82997E-20 |
| SPQ- Percep | -1.14 | 0.25 | 9.36 | 1.02677E-18 |
|  | 1.14 | 0.26 | 9.54 | 2.51555E-19 |
| **Self report frequency** | | | | |
|  | **t trait** | **p trait** | **t sex** | **p sex** |
| AQ- social | 2.29 | 0.02 | 11.26 | 2.75995E-25 |
| AQ- Switch | 0.28 | 0.78 | 11.00 | 2.31854E-24 |
| AQ- Detail | 0.64 | 0.52 | 11.03 | 1.87766E-24 |
| AQ- Comm | 1.19 | 0.23 | 11.03 | 1.80987E-24 |
| AQ- Imag | 1.53 | 0.13 | 11.04 | 1.64169E-24 |
| SPQ- Ideas | -1.39 | 0.17 | 10.73 | 2.166E-23 |
| SPQ- Constrict | -0.07 | 0.95 | 10.98 | 2.75402E-24 |
| SPQ- Eccentric | 1.56 | 0.12 | 10.95 | 3.54442E-24 |
| SPQ- Anxiety | 1.68 | 0.09 | 11.17 | 6.01766E-25 |
| SPQ- Magic | -2.27 | 0.02 | 10.16 | 2.08945E-21 |
| SPQ- Speech | 0.43 | 0.67 | 10.97 | 2.94436E-24 |
| SPQ- Percep | -0.86 | 0.39 | 10.90 | 5.46009E-24 |
|  | 0.86 | 0.39 | 11.04 | 1.62679E-24 |
| **Self report usage** | | | | |
|  | **t trait** | **p trait** | **t sex** | **p sex** |
| AQ- Social | -1.38 | 0.17 | -6.82 | 4.00094E-11 |
| AQ- Switch | 0.19 | 0.85 | -6.68 | 9.64936E-11 |
| AQ- Detail | -1.91 | 0.06 | -6.83 | 3.77794E-11 |
| AQ- Comm | -2.02 | 0.04 | -6.75 | 6.20419E-11 |
| AQ- Imag | 0.01 | 1.00 | -6.70 | 8.4876E-11 |
| SPQ- Ideas | -0.85 | 0.39 | -6.76 | 5.87755E-11 |
| SPQ- Constrict | -1.47 | 0.14 | -6.60 | 1.51157E-10 |
| SPQ- Eccentric | -2.75 | 0.01 | -6.63 | 1.30693E-10 |
| SPQ- Anxiety | -1.39 | 0.17 | -6.86 | 3.18905E-11 |
| SPQ- Magic | 0.93 | 0.35 | -6.26 | 1.13849E-09 |
| SPQ- Speech | -1.48 | 0.14 | -6.85 | 3.34411E-11 |
| SPQ- Percep | -0.70 | 0.49 | -6.74 | 6.65514E-11 |
|  | -1.73 | 0.09 | -6.82 | 4.14394E-11 |
| **Self report spare time** | | | | |
|  | **t trait** | **p trait** | **t sex** | **p sex** |
| AQ- Social | 1.75 | 0.08 | 9.26 | 2.12E-18 |
| AQ- Switch | -0.23 | 0.82 | 9.07 | 9.23E-18 |
| AQ- Detail | 1.01 | 0.31 | 9.15 | 5.01E-18 |
| AQ- Comm | 2.03 | 0.04 | 9.16 | 4.63E-18 |
| AQ- Imag | -0.35 | 0.73 | 9.09 | 7.54E-18 |
| SPQ- Ideas | -0.50 | 0.62 | 8.93 | 2.42E-17 |
| SPQ- Constrict | 0.29 | 0.77 | 9.05 | 1.02E-17 |
| SPQ- Eccentric | **2.89** | **0.004** | **9.05** | **1.06E-17** |
| SPQ- Anxiety | 2.13 | 0.03 | 9.39 | 8.16E-19 |
| SPQ- Magic | **-2.98** | **0.003** | **8.16** | **6.03E-15** |
| SPQ- Speech | 1.69 | 0.09 | 9.27 | 2.03E-18 |
| SPQ- Percep | -1.34 | 0.18 | 8.96 | 1.95E-17 |
|  | 1.33 | 0.18 | 9.18 | 4.02E-18 |

**Supplementary Table 6.** Video Game Genre Preferences in relation to AQ-Total, SPQ-Total, and SPQ-CogPer scores. Females = 207, Males = 144, Both = 351. F = females, M = males, B=both sexes.

| **Variables** | | **Scores**  **Mean ± SD (N)** | | | **T (df)** | **p value** |
| --- | --- | --- | --- | --- | --- | --- |
| **Action** | | | | | | |
| **AQ**: Total | F | Y | 21.50 | ± 5.88 (61) | 1.47(205) | 0.14 |
|  |  | N | 20.10 | ± 6.59 (146) |  |  |
|  | M | Y | 19.10 | ± 6.28 (111) | -1.27 (142) | 0.21 |
|  |  | N | 20.70 | ± 6.65 (33) |  |  |
|  | B | Y | 20.00 | ± 6.23 (172) | -0.34 (349) | 0.74 |
|  |  | N | 20.20 | ± 6.59 (179) |  |  |
| **SPQ:** Total | F | Y | 97.50 | ± 13.50 (61) | 1.44 (205) | 0.15 |
|  |  | N | 93.90 | ± 17.10 (146) |  |  |
|  | M | Y | 90.60 | ± 16.40 (111) | 0.97 (142) | 0.33 |
|  |  | N | 87.40 | ± 17.30 (33) |  |  |
|  | B | Y | 93.00 | ± 15.70 (172) | 0.18 (349) | 0.86 |
|  |  | N | 92.70 | ± 17.30(179) |  |  |
| **SPQ**:CogPer | F | Y | 39.60 | ± 8.68 (61) | 0.50 (205) | 0.62 |
|  |  | N | 39.00 | ± 8.24 (146) |  |  |
|  | M | Y | 35.60 | ± 9.01 (111) | 0.60 (142) | 0.55 |
|  |  | N | 34.50 | ± 8.55 (33) |  |  |
|  | B | Y | 37.00 | ± 9.07 (172) | -1.21 (349) | 0.23 |
|  |  | N | 38.20 | ± 8.46 (179) |  |  |
| **Platformer** | | | | | | |
| **AQ**: Total | F | Y | 20.20 | ± 6.13 (65) | -0.52 (205) | 0.60 |
|  |  | N | 20.70 | ± 6.55 (142) |  |  |
|  | M | Y | 17.50 | ± 7.28 (39) | **-2.34 (142)** | **0.02** |
|  |  | N | 20.20 | ± 5.87 (105) |  |  |
|  | B | Y | 19.20 | ± 6.67 (104) | -1.77 (349) | 0.08 |
|  |  | N | 20.50 | ± 6.26 (247) |  |  |
| **SPQ**:Total | F | Y | 96.00 | ± 17.70 (65) | 0.61 (205) | 0.54 |
|  |  | N | 94.50 | ± 15.50 (142) |  |  |
|  | M | Y | 89.30 | ± 19.10 (39) | -0.26 (142) | 0.79 |
|  |  | N | 90.10 | ± 15.60 (105) |  |  |
|  | B | Y | 93.50 | ± 18.40 (104) | 0.44 (349) | 0.66 |
|  |  | N | 92.60 | ± 15.70 (247) |  |  |
| **SPQ:**CogPer | F | Y | 39.70 | ± 9.37 (65) | 0.57 (205) | 0.57 |
|  |  | N | 39.00 | ± 7.88 (142) |  |  |
|  | M | Y | 34.50 | ± 9.64 (39) | -0.68 (142) | 0.50 |
|  |  | N | 35.70 | ± 8.62 (105) |  |  |
|  | B | Y | 37.70 | ± 9.75 (104) | 0.18 (349) | 0.86 |
|  |  | N | 37.60 | ± 8.34 (247) |  |  |
| **Puzzle** | | | | | | |
| **AQ**: Total | F | Y | 20.70 | ± 6.55 (205) | 0.42 (205) | 0.68 |
|  |  | N | 20.30 | ± 6.29 (205) |  |  |
|  | M | Y | 19.10 | ± 7.16 (43) | -0.52 (142) | 0.60 |
|  |  | N | 19.70 | ± 6.04 (101) |  |  |
|  | B | Y | 20.20 | ± 6.74 (150) | 0.34 (349) | 0.74 |
|  |  | N | 20.00 | ± 6.16 (201) |  |  |
| SPQ: TOTAL | F | Y | 97.50 | ± 14.90 (107) | **2.33 (205)** | **0.02** |
|  |  | N | 92.30 | ± 17.10 (100) |  |  |
|  | M | Y | 93.00 | ± 15.70 (43) | 1.51 (142) | 0.13 |
|  |  | N | 88.50 | ± 16.80 (101) |  |  |
|  | B | Y | 96.20 | ± 15.20 (150) | **3.31 (349)** | **0.001** |
|  |  | N | 90.40 | ± 17.10 (201) |  |  |
| SPQ-COG-PER | F | Y | 40.40 | ± 7.93 (107) | **2.16 (205)** | **0.03** |
|  |  | N | 37.90 | ± 8.65 (100) |  |  |
|  | M | Y | 36.30 | ± 9.37 (43) | 0.78 (142) | 0.44 |
|  |  | N | 35.00 | ± 8.69 (101) |  |  |
|  | B | Y | 39.20 | ± 8.54 (150) | **2.95 (349)** | **0.003** |
|  |  | N | 36.40 | ± 8.77 (201) |  |  |
| **RPG** | | | | | | |
| AQ: Total | F | Y | 23.00 | ± 7.33 (44) | **2.93 (205)** | **0.0038** |
|  |  | N | 19.80 | ± 5.99(163) |  |  |
|  | M | Y | 19.40 | ± 6.74 (74) | -0.27 (142) | 0.78 |
|  |  | N | 19.60 | ± 6.01 (70) |  |  |
|  | B | Y | 20.70 | ± 7.15 (118) | 1.27 (349) | 0.21 |
|  |  | N | 19.80 | ± 5.98 (233) |  |  |
| SPQ-TOTAL | F | Y | 95.80 | ± 18.20 (44) | 0.40 (205) | 0.69 |
|  |  | N | 94.70 | ± 15.70 (163) |  |  |
|  | M | Y | 90.60 | ± 15.80 (74) | 0.53 (142) | 0.60 |
|  |  | N | 89.10 | ± 17.40 (70) |  |  |
|  | B | Y | 92.50 | ± 16.90 (118) | -0.27 (349) | 0.79 |
|  |  | N | 93.00 | ± 16.40 (233) |  |  |
| SPQ-COG-PER | F | Y | 37.70 | ± 9.54 (44) | -1.34 (205) | 0.18 |
|  |  | N | 39.60 | ± 8.00 (163) |  |  |
|  | M | Y | 35.20 | ± 8.94 (74) | -0.28 (142) | 0.78 |
|  |  | N | 35.60 | ± 8.89 (70) |  |  |
|  | B | Y | 36.10 | ± 9.21 (118) | **-2.31 (349)** | **0.02** |
|  |  | N | 38.40 | ± 8.46 (233) |  |  |
| **Strategy** | | | | | | |
| AQ-Total | F | Y | 21.30 | ± 5.15(28) | 0.72 (205) | 0.47 |
|  |  | N | 20.40 | ± 6.59 (179) |  |  |
|  | M | Y | 19.60 | ± 6.99 (68) | 0.14 (142) | 0.89 |
|  |  | N | 19.40 | ± 5.82 (76) |  |  |
|  | B | Y | 20.10 | ± 6.53 (96) | -0.02 (349) | 0.98 |
|  |  | N | 20.10 | ± 6.37 (255) |  |  |
| SPQ-Total | F | Y | 93.60 | ± 11.60 (28) | -0.46 (205) | 0.64 |
|  |  | N | 95.20 | ± 16.80 (179) |  |  |
|  | M | Y | 92.50 | ± 17.20 (68) | 1.86 (142) | 0.07 |
|  |  | N | 87.50 | ± 15.70 (76) |  |  |
|  | B | Y | 92.90 | ± 15.70 (96) | -0.01 (349) | 0.99 |
|  |  | N | 92.90 | ± 16.90 (255) |  |  |
| SPQ-CogPer | F | Y | 37.50 | ± 7.08 (28) | -1.12 (205) | 0.27 |
|  |  | N | 39.40 | ± 8.53 (179) |  |  |
|  | M | Y | 36.40 | ± 9.15 (68) | 1.32 (142) | 0.19 |
|  |  | N | 34.40 | ± 8.60 (76) |  |  |
|  | B | Y | 36.70 | ± 8.58 (96) | -1.16 (349) | 0.25 |
|  |  | N | 37.90 | ± 8.83 (255) |  |  |
| **Sports** | | | | | | |
| AQ-Total | F | Y | 18.60 | ± 4.95 (29) | -1.75 (205) | 0.08 |
|  |  | N | 20.80 | ± 6.57 (178) |  |  |
|  | M | Y | 17.90 | ± 5.79 (44) | **-2.06 (142)** | **0.04** |
|  |  | N | 20.20 | ± 6.51 (100) |  |  |
|  | B | Y | 18.20 | ± 5.45 (73) | **-2.94 (349)** | **0.004** |
|  |  | N | 20.60 | ± 6.55 (278) |  |  |
| SPQ-Total | F | Y | 91.10 | ± 14.30 (29) | -1.40 (205) | 0.16 |
|  |  | N | 95.60 | ± 16.40 (178) |  |  |
|  | M | Y | 89.90 | ± 20.20 (44) | 0.03 (142) | 0.98 |
|  |  | N | 89.80 | ± 14.80 (100) |  |  |
|  | B | Y | 90.40 | ± 18.00 (73) | -1.45 (349) | 0.15 |
|  |  | N | 93.50 | ± 16.10 (278) |  |  |
| SPQ-CogPer | F | Y | 37.90 | ± 8.13 (29) | -0.89 (205) | 0.38 |
|  |  | N | 39.40 | ± 8.40 (178) |  |  |
|  | M | Y | 36.60 | ± 10.90 (44) | 1.14 (142) | 0.26 |
|  |  | N | 34.80 | ± 7.81 (100) |  |  |
|  | B | Y | 37.10 | ± 9.88 (73) | -0.52 (349) | 0.60 |
|  |  | N | 37.70 | ± 8.47 (278) |  |  |
| **Racing** | | | | | | |
| AQ: Total | F | Y | 20.60 | ± 5.47 (44) | 0.12 (205) | 0.91 |
|  |  | N | 20.50 | ± 6.65 (163) |  |  |
|  | M | Y | 20.40 | ± 6.18 (32) | 0.95 (142) | 0.34 |
|  |  | N | 19.20 | ± 6.43 (112) |  |  |
|  | B | Y | 20.60 | ± 5.74 (76) | 0.68 (349) | 0.49 |
|  |  | N | 20.00 | ± 6.58 (275) |  |  |
| SPQ-Total | F | Y | 97.10 | ± 14.90 (44) | 0.97 (205) | 0.33 |
|  |  | N | 94.40 | ± 16.50 (163) |  |  |
|  | M | Y | 94.80 | ± 13.20 (32) | 1.95 (142) | 0.053 |
|  |  | N | 88.40 | ± 17.20 (112) |  |  |
|  | B | Y | 96.10 | ± 14.20 (76) | **1.95 (349)** | **0.05** |
|  |  | N | 92.00 | ± 17.00 (275) |  |  |
| SPQ**:**CogPer | F | Y | 40.30 | ± 8.29 (44) | 1.00 (205) | 0.32 |
|  |  | N | 38.90 | ± 8.38 (163) |  |  |
|  | M | Y | 39.10 | ± 8.42 (32) | **2.73 (142)** | **0.007** |
|  |  | N | 34.30 | ± 8.77 (112) |  |  |
|  | B | Y | 39.80 | ± 8.31 (76) | **2.45 (349)** | **0.015** |
|  |  | N | 37.00 | ± 8.81 (275) |  |  |
| **Construction** | | | | | | |
| AQ: Total | F | Y | 22.40 | ± 6.48 (65) | **2.85 (205)** | **0.0049** |
|  |  | N | 19.70 | ± 6.22 (142) |  |  |
|  | M | Y | 19.70 | ± 6.93 (57) | 0.32 (142) | 0.75 |
|  |  | N | 19.40 | ± 6.02 (87) |  |  |
|  | B | Y | 21.10 | ± 6.80 (122) | **2.19 (349)** | **0.03** |
|  |  | N | 19.60 | ± 6.13 (229) |  |  |
| SPQ:Total | F | Y | 95.80 | ± 16.50 (65) | 0.48 (205) | 0.63 |
|  |  | N | 94.60 | ± 16.10 (142) |  |  |
|  | M | Y | 89.20 | ± 17.40 (57) | -0.39 (142) | 0.70 |
|  |  | N | 90.30 | ± 16.10 (87) |  |  |
|  | B | Y | 92.70 | ± 17.20 (122) | -0.14 (349) | 0.89 |
|  |  | N | 92.00 | ± 16.20 (229) |  |  |
| SQ:Cog-Per | F | Y | 38.20 | ± 8.23 (65) | -1.19 (205) | 0.24 |
|  |  | N | 39.60 | ± 8.41 (142) |  |  |
|  | M | Y | 34.00 | ± 9.00 (57) | -1.46 (142) | 0.15 |
|  |  | N | 36.20 | ± 8.75 (87) |  |  |
|  | B | Y | 36.20 | ± 8.81 (122) | **-2.17 (349)** | **0.03** |
|  |  | N | 38.30 | ± 8.68 (229) |  |  |
| **Social Simulation** | | | | | | |
| AQ: Total | F | Y | 22.20 | ± 6.48 (76) | 1.13 (205) | 0.26 |
|  |  | N | 20.10 | ± 6.36 (131) |  |  |
|  | M | Y | 20.10 | ± 7.40 (20) | 0.42 (142) | 0.68 |
|  |  | N | 19.40 | ± 6.22 (124) |  |  |
|  | B | Y | 20.90 | ± 6.66 (96) | 1.52 (349) | 0.13 |
|  |  | N | 19.80 | ± 6.29 (255) |  |  |
| SPQ:Total | F | Y | 96.80 | ± 17.70 (76) | 1.23 (205) | 0.22 |
|  |  | N | 93.90 | ± 15.20 (131) |  |  |
|  | M | Y | 91.00 | ± 19.00 (20) | 0.35 (142) | 0.73 |
|  |  | N | 89.70 | ± 16.20 (124) |  |  |
|  | B | Y | 95.60 | ± 18.00 (96) | **1.90 (349)** | **0.058** |
|  |  | N | 91.80 | ± 15.80 (255) |  |  |
| **SPQ:**CogPer | F | Y | 39.40 | ± 9.08 (76) | 0.29 (205) | 0.77 |
|  |  | N | 39.00 | ± 7.94 (131) |  |  |
|  | M | Y | 35.80 | ± 10.10 (20) | 0.23 (142) | 0.82 |
|  |  | N | 35.30 | ± 8.72 (124) |  |  |
|  | B | Y | 38.60 | ± 9.36 (96) | 1.36 (349) | 0.18 |
|  |  | N | 37.20 | ± 8.52 (255) |  |  |
| **Idle** | | | | | | |
| AQ: Total | F | Y | 21.90 | ± 5.22 (20) | 1.02 (205) | 0.31 |
|  |  | N | 20.40 | ± 6.52 (187) |  |  |
|  | M | Y | 21.90 | ± 5.83 (15) | 1.53 (142) | 0.13 |
|  |  | N | 19.20 | ± 6.40 (129) |  |  |
|  | B | Y | 21.90 | ± 5.41 (35) | 1.75 (349) | 0.08 |
|  |  | N | 19.90 | ± 6.48 (316) |  |  |
| SPQ:Total | F | Y | 94.50 | ± 15.80 (20) | -0.15 (205) | 0.88 |
|  |  | N | 95.00 | ± 16.30 (187) |  |  |
|  | M | Y | 91.60 | ± 17.60 (15) | 0.43(142) | 0.67 |
|  |  | N | 89.70 | ± 16.50 (129) |  |  |
|  | B | Y | 93.20 | ± 16.40 (35) | 0.14 (349) | 0.89 |
|  |  | N | 92.80 | ± 16.60 (316) |  |  |
| SPQ:CogPer | F | Y | 38.90 | ± 7.90 (20) | -0.18 (205) | 0.86 |
|  |  | N | 39.20 | ± 8.43 187() |  |  |
|  | M | Y | 34.00 | ± 9.77 (15) | -0.63 (142) | 0.53 |
|  |  | N | 35.50 | ± 8.80 (129) |  |  |
|  | B | Y | 36.80 | ± 8.95 (35) | -0.60 (349) | 0.55 |
|  |  | N | 37.70 | ± 8.76 (316) |  |  |

**Supplementary Table 7.** Stepwise Regression Analyses of AQ and SPQ scores in relation to Video Game Genre Preferences

| **Puzzle** | | | | | |
| --- | --- | --- | --- | --- | --- |
| # | Estimate | Std. Error | z value | Pr(>\|z\|) | Signficance |
| #(Intercept) | -1.51323 | 0.48524 | -3.118 | 0.001818 | ** |
| #AQ-Imag | -0.17066 | 0.07576 | -2.253 | 0.024287 | * |
| #SPQ- Eccentric | 0.13749 | 0.03181 | 4.322 | 1.54E-05 | *** |
| #SPQ-Magic | 0.05395 | 0.03135 | 1.721 | 0.085245 |  |
| #sexM | -0.9549 | 0.24713 | -3.864 | 0.000112 | *** |
|  |  |  |  |  |  |
| **Action** | | | | | |
| #(Intercept) | -1.89453 | 0.41886 | -4.523 | 6.09E-06 | *** |
| #AQ-Social | -0.08123 | 0.05261 | -1.544 | 0.12259 |  |
| #SPQ- Eccentric | 0.11546 | 0.03516 | 3.284 | 0.00102 | ** |
| #sexM | 2.08247 | 0.25588 | 8.139 | 4.00E-16 | *** |
|  |  |  |  |  |  |
| **Platformer** | | | | | |
| #(Intercept) | -1.33622 | 0.37984 | -3.518 | 0.000435 | *** |
| #AQ-Social | -0.09942 | 0.05103 | -1.948 | 0.051408 |  |
| #SPQ- Eccentric | 0.0698 | 0.03264 | 2.138 | 0.032507 | * |
|  |  |  |  |  |  |
| **RPG** | | | | | |
| #(Intercept) | -1.76468 | 0.56657 | -3.115 | 0.00184 | ** |
| #AQ-Social | 0.10701 | 0.05107 | 2.095 | 0.03614 | * |
| #SPQ- Ideas | -0.06357 | 0.02866 | -2.218 | 0.02656 | * |
| #SPQ- Eccentric | 0.1097 | 0.03725 | 2.945 | 0.00323 | ** |
| #sexM | 1.38089 | 0.25022 | 5.519 | 3.41E-08 | *** |
|  |  |  |  |  |  |
| **Strategy** | | | | | |
| #(Intercept) | -2.33546 | 0.5477 | -4.264 | 2.01E-05 | *** |
| #AQ- Switch | -0.12767 | 0.07105 | -1.797 | 0.07235 |  |
| #SPQ- Eccentric | 0.10477 | 0.03723 | 2.814 | 0.00489 | ** |
| #sexM | 1.73843 | 0.26751 | 6.499 | 8.11E-11 | *** |
|  |  |  |  |  |  |
| **Sports** | | | | | |
| #(Intercept) | -1.80606 | 0.66455 | -2.718 | 0.006574 | ** |
| #AQ- Switch | -0.13697 | 0.079 | -1.734 | 0.082961 |  |
| #AQ- Comm | -0.22088 | 0.07947 | -2.779 | 0.005447 | ** |
| #SPQ- Constrict | 0.04847 | 0.03117 | 1.555 | 0.119915 |  |
| #SPQ- Magic | 0.05772 | 0.03728 | 1.548 | 0.121599 |  |
| #sexM | 1.09101 | 0.28916 | 3.773 | 0.000161 | *** |
|  |  |  |  |  |  |
| **Racing** | | | | | |
| #(Intercept) | -2.38204 | 0.46174 | -5.159 | 2.49E-07 | *** |
| #AQ- Detail | 0.09746 | 0.06138 | 1.588 | 0.1124 |  |
| #SPQ- Magic | 0.0611 | 0.03403 | 1.796 | 0.0726 |  |
|  |  |  |  |  |  |
| **Construction** | | | | | |
| #(Intercept) | -0.72016 | 0.62183 | -1.158 | 0.2468 |  |
| #AQ-Social | 0.11016 | 0.04741 | 2.324 | 0.0201 | * |
| #SPQ- Ideas | -0.05226 | 0.02753 | -1.898 | 0.0577 |  |
| #SPQ- Magic | -0.06593 | 0.0334 | -1.974 | 0.0484 | * |
| #SPQ- Speech | 0.08917 | 0.03895 | 2.289 | 0.0221 | * |
|  |  |  |  |  |  |
| **Social Simulation** | | | | | |
| #(Intercept) | -1.28107 | 0.5828 | -2.198 | 0.0279 | * |
| #AQ- Switch | 0.10863 | 0.06891 | 1.576 | 0.115 |  |
| #AQ- Detail | -0.09946 | 0.05649 | -1.761 | 0.0783 |  |
| #SPQ- Eccentric | 0.05913 | 0.03457 | 1.71 | 0.0872 |  |
| #sexM | -1.33153 | 0.2858 | -4.659 | 3.18E-06 | *** |
|  |  |  |  |  |  |
| **Idle** | | | | | |
| #(Intercept) | -3.50343 | 0.66148 | -5.296 | 1.18E-07 | *** |
| #social | 0.1517 | 0.09335 | 1.625 | 0.1041 |  |
| #AQ- Switch | 0.21382 | 0.11318 | 1.889 | 0.0589 |  |
| #AQ- Comm | -0.15725 | 0.11249 | -1.398 | 0.1622 |  |
|  |  |  |  |  |  |
| **Game Type Choice by AQ-Total, SPQ-Total, SPQ-CogPer and sex** | | | | |  |
| **Puzzle** | | | | | |
| # | Estimate | Std. Error | z-value | Pr(>\|z\|) |  |
| #(Intercept) | -0.118893 | 0.467646 | -0.254 | 0.799 |  |
| #AQ-total | 0.009098 | 0.02178 | 0.418 | 0.676 |  |
| #sexM | -0.4451 | 0.746599 | -0.596 | 0.551 |  |
| #AQ- total:sexM | -0.024064 | 0.036038 | -0.668 | 0.504 |  |
|  |  |  |  |  |  |
| **Action** |  |  |  |  |  |
| #(Intercept) | -1.60712 | 0.53062 | -3.029 | 0.00246 | ** |
| #AQ- total | 0.0353 | 0.02409 | 1.466 | 0.14276 |  |
| #sexM | 3.62131 | 0.86051 | 4.208 | 2.57E-05 | *** |
| #AQ- total:sexM | -0.0755 | 0.03989 | -1.893 | 0.0584 |  |
|  |  |  |  |  |  |
| **Platformer** | | | | | |
| #(Intercept) | -0.53114 | 0.50056 | -1.061 | 0.289 |  |
| #AQ-Total | -0.01226 | 0.0235 | -0.522 | 0.602 |  |
| #sexM | 0.86546 | 0.77838 | 1.112 | 0.266 |  |
| #AQ- total:sexM | -0.05799 | 0.03885 | -1.493 | 0.136 |  |
|  |  |  |  |  |  |
| **RPG** | | | | | |
| #(Intercept) | -2.99672 | 0.64555 | -4.642 | 3.45E-06 | *** |
| #AQ- Total | 0.07881 | 0.02794 | 2.82 | 0.004801 | ** |
| #sexM | 3.19316 | 0.84072 | 3.798 | 0.000146 | *** |
| #AQ- total:sexM | -0.08603 | 0.03835 | -2.243 | 0.024866 | * |
|  |  |  |  |  |  |
| **Strategy** | | | | | |
| #(Intercept) | -2.3316 | 0.70327 | -3.315 | 0.000915 | *** |
| #AQ- Total | 0.02285 | 0.03177 | 0.719 | 0.472003 |  |
| #sexM | 2.1467 | 0.88606 | 2.423 | 0.015404 | * |
| #AQ- total:sexM | -0.01907 | 0.04123 | -0.463 | 0.643708 |  |
|  |  |  |  |  |  |
| **Sports** | | | | | |
| #(Intercept) | -0.702094 | 0.647946 | -1.084 | 0.2786 |  |
| #AQ- Total | -0.05648 | 0.032573 | -1.734 | 0.0829 |  |
| #sexM | 1.01263 | 0.868298 | 1.166 | 0.2435 |  |
| #AQ- total:sexM | -0.002963 | 0.043931 | -0.067 | 0.9462 |  |
|  |  |  |  |  |  |
| **Racing** | | | | | |
| # | Estimate | Std. Error | z-value | Pr(>\|z\|) |  |
| #(Intercept) | -1.374363 | 0.572545 | -2.4 | 0.0164 | * |
| #AQ- Total | 0.003154 | 0.026559 | 0.119 | 0.9055 |  |
| #sexM | -0.479859 | 0.884456 | -0.543 | 0.5874 |  |
| #AQ- total:sexM | 0.027174 | 0.041512 | 0.655 | 0.5127 |  |
|  |  |  |  |  |  |
| **Construction** | | | | | |
| #(Intercept) | -2.19981 | 0.54799 | -4.014 | 5.96E-05 | *** |
| #AQ-Total | 0.06752 | 0.02451 | 2.755 | 0.00588 | ** |
| #sexM | 1.60969 | 0.77802 | 2.069 | 0.03855 | * |
| #AQ- total:sexM | -0.05896 | 0.03636 | -1.622 | 0.10488 |  |
|  |  |  |  |  |  |
| **Social Simulation** | | | | | |
| #(Intercept) | -1.072182 | 0.493229 | -2.174 | 0.0297 | * |
| #AQ- total | 0.025556 | 0.022688 | 1.126 | 0.26 |  |
| #sexM | -1.069396 | 0.937451 | -1.141 | 0.254 |  |
| #AQ- total:sexM | -0.009486 | 0.044317 | -0.214 | 0.8305 |  |
|  |  |  |  |  |  |
| **Idle** | | | | | |
| #(Intercept) | -3.02785 | 0.8354 | -3.624 | 0.00029 | *** |
| #AQ- total | 0.0375 | 0.03686 | 1.018 | 0.30886 |  |
| #sexM | -0.51805 | 1.30974 | -0.396 | 0.69245 |  |
| #AQ- total:sexM | 0.03031 | 0.05814 | 0.521 | 0.60206 |  |
|  |  |  |  |  |  |
| **Puzzle** | | | | | |
| #(Intercept) | -1.35935 | 0.68802 | -1.976 | 0.0482 | * |
| #SPQ- Cog-Per | 0.03645 | 0.01721 | 2.118 | 0.0341 | * |
| #sexM | -0.07061 | 1.02704 | -0.069 | 0.9452 |  |
| #SPQ- Cog-Per:sexM | -0.02028 | 0.02687 | -0.755 | 0.4504 |  |
|  |  |  |  |  |  |
| **Action** | | | | | |
| #(Intercept) | -1.234576 | 0.741326 | -1.665 | 0.0958 |  |
| #SPQ- Cog-Per | 0.009206 | 0.018395 | 0.5 | 0.6168 |  |
| #sexM | 1.967776 | 1.096967 | 1.794 | 0.0728 |  |
| #SPQ- Cog-Per:sexM | 0.004473 | 0.029086 | 0.154 | 0.8778 |  |
|  |  |  |  |  |  |
| **Platformer** | | | | | |
| #(Intercept) | -1.18562 | 0.72866 | -1.627 | 0.104 |  |
| #SPQ- Cog-Per | 0.01028 | 0.01808 | 0.569 | 0.57 |  |
| #sexM | 0.70731 | 1.05739 | 0.669 | 0.504 |  |
| #SPQ- Cog-Per:sexM | -0.02487 | 0.02795 | -0.89 | 0.374 |  |
|  |  |  |  |  |  |
| **RPG** | | | | | |
| #(Intercept) | -0.26627 | 0.79159 | -0.336 | 0.737 |  |
| #SPQ- Cog-Per | -0.027 | 0.02028 | -1.331 | 0.183 |  |
| #sexM | 0.51302 | 1.04825 | 0.489 | 0.625 |  |
| #SPQ- Cog-Per:sexM | 0.0216 | 0.02768 | 0.78 | 0.435 |  |
|  |  |  |  |  |  |
| **Strategy** | | | | | |
| #(Intercept) | -0.82325 | 0.93266 | -0.883 | 0.3774 |  |
| #SPQ- Cog-Per | -0.02681 | 0.02407 | -1.114 | 0.2653 |  |
| #sexM | -0.17698 | 1.16529 | -0.152 | 0.8793 |  |
| #SPQ- Cog-Per:sexM | 0.05191 | 0.03074 | 1.689 | 0.0913 |  |
|  |  |  |  |  |  |
| **Sports** | | | | | |
| #(Intercept) | -1.00003 | 0.92707 | -1.079 | 0.281 |  |
| #SPQ- Cog-Per | -0.02108 | 0.02375 | -0.888 | 0.375 |  |
| #sexM | -0.65724 | 1.20225 | -0.547 | 0.585 |  |
| #SPQ- Cog-Per:sexM | 0.04449 | 0.03145 | 1.415 | 0.157 |  |
|  |  |  |  |  |  |
| **Racing** | | | | | |
| # | Estimate | Std. Error | z-value | Pr(>\|z\|) |  |
| #(Intercept) | -2.13582 | 0.84976 | -2.513 | 0.012 | * |
| #SPQ- Cog-Per | 0.02087 | 0.02081 | 1.003 | 0.316 |  |
| #sexM | -1.4326 | 1.26808 | -1.13 | 0.259 |  |
| #SPQ- Cog-Per:sexM | 0.04224 | 0.03193 | 1.323 | 0.186 |  |
|  |  |  |  |  |  |
| **Construction** | | | | | |
| #(Intercept) | 0.04839 | 0.71066 | 0.068 | 0.946 |  |
| #SPQ- Cog-Per | -0.02133 | 0.01798 | -1.187 | 0.235 |  |
| #sexM | 0.53148 | 1.00207 | 0.53 | 0.596 |  |
| #SPQ- Cog-Per:sexM | -0.00721 | 0.02662 | -0.271 | 0.787 |  |
|  |  |  |  |  |  |
| **Social Simulation** | | | | | |
| #(Intercept) | -0.741634 | 0.695537 | -1.066 | 0.286 |  |
| #SPQ- Cog-Per | 0.005027 | 0.017328 | 0.29 | 0.772 |  |
| #sexM | -1.31013 | 1.218963 | -1.075 | 0.282 |  |
| #SPQ- Cog-Per:sexM | 0.001364 | 0.032244 | 0.042 | 0.966 |  |
|  |  |  |  |  |  |
| **Idle** | | | | | |
| #(Intercept) | -2.035076 | 1.117093 | -1.822 | 0.0685 |  |
| #SPQ- Cog-Per | -0.005132 | 0.028084 | -0.183 | 0.855 |  |
| #sexM | 0.566718 | 1.567005 | 0.362 | 0.7176 |  |
| #SPQ- Cog-Per:sexM | -0.014528 | 0.041949 | -0.346 | 0.7291 |  |
|  |  |  |  |  |  |
| **Puzzle** | | | | | |
| #(Intercept) | -1.874173 | 0.863866 | -2.17 | 0.03 | * |
| #SPQ- total | 0.020456 | 0.008978 | 2.279 | 0.0227 | * |
| #sexM | -0.532091 | 1.367703 | -0.389 | 0.6972 |  |
| #SPQ- total:sexM | -0.003358 | 0.0145 | -0.232 | 0.8168 |  |
|  |  |  |  |  |  |
| **Action** | | | | | |
| #(Intercept) | -2.212225 | 0.952465 | -2.323 | 0.0202 | * |
| #SPQ- total | 0.013994 | 0.009745 | 1.436 | 0.151 |  |
| #sexM | 2.388551 | 1.437268 | 1.662 | 0.0965 |  |
| #SPQ- total:sexM | -0.002346 | 0.015458 | -0.152 | 0.8794 |  |
|  |  |  |  |  |  |
| **Platformer** | | | | | |
| # | Estimate | Std. Error | z-value | Pr(>\|z\|) |  |
| #(Intercept) | -1.327172 | 0.905632 | -1.465 | 0.143 |  |
| #SPQ- total | 0.00573 | 0.00935 | 0.613 | 0.54 |  |
| #sexM | 0.605466 | 1.372321 | 0.441 | 0.659 |  |
| #SPQ- total:sexM | -0.008726 | 0.01469 | -0.594 | 0.552 |  |
|  |  |  |  |  |  |
| **RPG** | | | | | |
| #(Intercept) | -1.717973 | 1.02738 | -1.672 | 0.0945 |  |
| #SPQ- total | 0.004286 | 0.010598 | 0.404 | 0.6859 |  |
| #sexM | 1.290069 | 1.381488 | 0.934 | 0.3504 |  |
| #SPQ- total:sexM | 0.001096 | 0.014649 | 0.075 | 0.9404 |  |
|  |  |  |  |  |  |
| **Strategy** | | | | | |
| # | Estimate | Std. Error | z-value | Pr(>\|z\|) |  |
| #(Intercept) | -1.307872 | 1.185861 | -1.103 | 0.27 |  |
| #SPQ- total | -0.005797 | 0.012448 | -0.466 | 0.641 |  |
| #sexM | -0.512669 | 1.523766 | -0.336 | 0.737 |  |
| #SPQ- total:sexM | 0.024785 | 0.016247 | 1.526 | 0.127 |  |
|  |  |  |  |  |  |
| **Sports** | | | | | |
| #(Intercept) | -0.23329 | 1.13373 | -0.206 | 0.837 |  |
| #SPQ- total | -0.01693 | 0.01215 | -1.393 | 0.164 |  |
| #sexM | -0.61372 | 1.51215 | -0.406 | 0.685 |  |
| #SPQ- total:sexM | 0.01722 | 0.01636 | 1.053 | 0.292 |  |
|  |  |  |  |  |  |
| **Racing** | | | | | |
| #(Intercept) | -2.3093 | 1.05286 | -2.193 | 0.0283 | * |
| #SPQ- total | 0.01044 | 0.01076 | 0.97 | 0.3321 |  |
| #sexM | -1.2014 | 1.61185 | -0.745 | 0.4561 |  |
| #SPQ- total:sexM | 0.01419 | 0.01679 | 0.845 | 0.398 |  |
|  |  |  |  |  |  |
| **Construction** | | | | | |
| #(Intercept) | -1.210622 | 0.902417 | -1.342 | 0.18 |  |
| #SPQ- total | 0.004509 | 0.009327 | 0.483 | 0.629 |  |
| #sexM | 1.147236 | 1.302986 | 0.88 | 0.379 |  |
| #SPQ- total:sexM | -0.008514 | 0.013905 | -0.612 | 0.54 |  |
|  |  |  |  |  |  |
| **Social Simulation** | | | | | |
| #(Intercept) | -1.61399 | 0.884194 | -1.825 | 0.0679 |  |
| #SPQ- total | 0.011215 | 0.009108 | 1.231 | 0.2182 |  |
| #sexM | -0.673131 | 1.61886 | -0.416 | 0.6776 |  |
| #SPQ- total:sexM | -0.006096 | 0.017282 | -0.353 | 0.7243 |  |
|  |  |  |  |  |  |
| **Idle** | | | | | |
| #(Intercept) | -2.029095 | 1.389358 | -1.46 | 0.144 |  |
| #SPQ- total | -0.002177 | 0.014489 | -0.15 | 0.881 |  |
| #sexM | -0.776283 | 2.081821 | -0.373 | 0.709 |  |
| #SPQ- total:sexM | 0.009389 | 0.022107 | 0.425 | 0.671 |  |

* < denotes significance at <0.05

** < denotes significance at < 0.01

*** denotes significance at < 0.001

**Supplementary Table 8**. Gaming Motivations in relation to AQ-Total, SPQ-Total and SPQ-CogPer scores. Sample sizes for Females = 207, Males = 144, and Both Sexes = 351)

| Variables | | Scores  Mean ± SD (N) | | | T (df) | p value |
| --- | --- | --- | --- | --- | --- | --- |
| **Social Interaction** | | | | | | |
| AQ- Total | F | Y | 20.97 | ± 6.27 (61) | 0.67 (116) | 0.51 |
|  |  | N | 20.32 | ± 6.48 (146) |  |  |
|  | M | Y | 19.13 | ± 6.11 (76) | -0.71 (136) | 0.48 |
|  |  | N | 19.90 | ± 6.68 (68) |  |  |
|  | B | Y | 19.50 | ± 6.22 (137) | -0.34 (300) | 0.73 |
|  |  | N | 20.19 | ± 6.53 (214) |  |  |
| SPQ-Total | F | Y | 95.62 | ± 14.60 (61) | 0.40 (129) | 0.69 |
|  |  | N | 94.69 | ± 16.86 (146) |  |  |
|  | M | Y | 89.68 | ± 17.03 (76) | -0.13 (141) | 0.90 |
|  |  | N | 90.04 | ± 16.19 (68) |  |  |
|  | B | Y | 92.33 | ± 16.21 (137) | -0.49 (297) | 0.62 |
|  |  | N | 93.22 | ± 16.76 (214) |  |  |
| SPQ:CogPer | F | Y | 39.31 | ± 8.10 (61) | 0.16 (118) | 0.88 |
|  |  | N | 39.12 | ± 8.49 (146) |  |  |
|  | M | Y | 34.90 | ± 8.62 (76) | -0.67 (138) | 0.50 |
|  |  | N | 35.90 | ± 9.21 (68) |  |  |
|  | B | Y | 36.86 | ± 8.65 (137) | -1.29 (294) | 0.20 |
|  |  | N | 38.09 | ± 8.83 (214) |  |  |
| **Stress Relief** | | | | | | |
| AQ-Total | F | Y | 21.39 | ± 6.29 (131) | **2.62 (155)** | **0.0099** |
|  |  | N | 19.00 | ± 6.37 (76) |  |  |
|  | M | Y | 91.43 | ± 16.13 (104) | 1.80 (67) | 0.07 |
|  |  | N | 85.75 | ± 17.25 (40) |  |  |
|  | B | Y | 20.65 | ± 6.25 (235) | **2.29 (218)** | **0.02** |
|  |  | N | 18.97 | ± 6.60 (116) |  |  |
| SPQ-Total | F | Y | 96.17 | ± 16.42 (131) | 1.42 (163) | 0.16 |
|  |  | N | 92.90 | ± 15.70 (76) |  |  |
|  | M | Y | 91.43 | ± 16.13 (104) | 1.80 (67) | 0.08 |
|  |  | N | 85.75 | ± 17.25 (40) |  |  |
|  | B | Y | 94.07 | ± 16.43 (235) | **1.94 (228)** | 0.053 |
|  |  | N | 90.43 | ± 16.53 (116) |  |  |
| SPQ-CogPer | F | Y | 39.26 | ± 8.61 (131) | 0.20 (167) | 0.84 |
|  |  | N | 39.03 | ± 7.96 (76) |  |  |
|  | M | Y | 36.35 | ± 8.88 (104) | **2.20 (74)** | **0.03** |
|  |  | N | 32.83 | ± 8.5 (40) |  |  |
|  | B | Y | 37.97 | ± 8.83 (235) | 1.10 (234) | 0.27 |
|  |  | N | 36.89 | ± 8.63 (116) |  |  |
| **Skill Development** | | | | | | |
| AQ-Total | F | Y | 22.18 | ± 6.00 (33) | 1.73 (47) | 0.09 |
|  |  | N | 20.20 | ± 6.46 (174) |  |  |
|  | M | Y | 92.90 | ± 15.25 (50) | 1.67 (110) | 0.10 |
|  |  | N | 88.23 | ± 17.11 (94) |  |  |
|  | B | Y | 21.28 | ± 5.98 (83) | **2.02 (147)** | **0.045** |
|  |  | N | 19.73 | ± 6.50 (268) |  |  |
| SPQ-Total | F | Y | 98.21 | ± 15.09 (33) | 1.33 (47) | 0.20 |
|  |  | N | 94.35 | ± 16.37 (174) |  |  |
|  | M | Y | 92.90 | ± 15.25 (50) | 1.67 (110) | 0.10 |
|  |  | N | 88.23 | ± 17.11 (94) |  |  |
|  | B | Y | 95.01 | ± 15.32 (83) | 1.42 (149) | 0.16 |
|  |  | N | 92.21 | ± 16.86 (268) |  |  |
| SPQ-CogPer | F | Y | 40.33 | ± 8.52 (33) | 0.86 (44) | 0.40 |
|  |  | N | 38.95 | ± 8.34 (174) |  |  |
|  | M | Y | 37.28 | ± 8.59 (50) | **1.92 (103)** | 0.057 |
|  |  | N | 34.35 | ± 8.92 (94) |  |  |
|  | B | Y | 38.49 | ± 8.64 (83) | 1.06 (139) | 0.30 |
|  |  | N | 37.34 | ± 8.81 (268) |  |  |
| **Adrenaline Rush** | | | | | | |
| AQ-Total | F | Y | 21.63 | ± 5.16 (46) | 1.55 (93) | 0.12 |
|  |  | N | 20.19 | ± 6.70 (161) |  |  |
|  | M | Y | 90.40 | ± 17.93 (58) | 0.31 (111) | 0.75 |
|  |  | N | 89.49 | ± 15.71 (86) |  |  |
|  | B | Y | 37.60 | ± 8.95 (104) | -0.02 (189) | 0.98 |
|  |  | N | 37.62 | ± 8.71 (247) |  |  |
| SPQ-Total | F | Y | 95.87 | ± 14.21(46) | 0.47 (84) | 0.64 |
|  |  | N | 94.71 | ± 16.76 (161) |  |  |
|  | M | Y | 90.40 | ± 17.93 (58) | 0.31 (111) | 0.75 |
|  |  | N | 89.49 | ± 15.71 (86) |  |  |
|  | B | Y | 92.82 | ± 16.54 (104) | -0.04 (194) | 0.97 |
|  |  | N | 92.89 | ± 16.55 (247) |  |  |
| SPQ-CogPer | F | Y | 39.41 | ± 8.21 (46) | 0.22 (74) | 0.82 |
|  |  | N | 39.11 | ± 8.43 (161) |  |  |
|  | M | Y | 36.16 | ± 9.31 (58) | 0.86 (116) | 0.39 |
|  |  | N | 34.84 | ± 8.60 (86) |  |  |
|  | B | Y | 37.60 | ± 8.95 (104) | -0.02 (189) | 0.98 |
|  |  | N | 37.62 | ± 8.71 (247) |  |  |
| **Escape** | | | | | | |
| AQ-Total | F | Y | 20.89 | ± 6.66 (116) | 0.96 (200) | 0.34 |
|  |  | N | 20.03 | ± 6.07 (91) |  |  |
|  | M | Y | 19.58 | ± 6.42 (91) | 0.22 (110) | 0.83 |
|  |  | N | 19.34 | ± 6.36 (53) |  |  |
|  | B | Y | 20.31 | ± 6.57 (207) | 0.78(320) | 0.44 |
|  |  | N | 19.78 | ± 6.17 (144) |  |  |
| SPQ-Total | F | Y | 94.66 | ± 15.99 (116) | -0.31 (190) | 0.76 |
|  |  | N | 95.36 | ± 16.54 (91) |  |  |
|  | M | Y | 91.90 | ± 17.27(91) | **2.04 (122)** | **0.04** |
|  |  | N | 86.34 | ± 14.83 (53) |  |  |
|  | B | Y | 93.44 | ± 16.58 (207) | 0.78 (309) | 0.43 |
|  |  | N | 92.04 | ± 16.47 (144) |  |  |
| SPQ-CogPer | F | Y | 38.31 | ± 8.55 (116) | -1.70 (198) | 0.09 |
|  |  | N | 40.28 | ± 8.03 (91) |  |  |
|  | M | Y | 36.13 | ± 9.07 (91) | 1.38 (115) | 0.17 |
|  |  | N | 34.06 | ± 8.48 (53) |  |  |
|  | B | Y | 37.35 | ± 8.83 (207) | -0.67 (310) | 0.50 |
|  |  | N | 37.99 | ± 8.70 (144) |  |  |
| **Fantasy** | | | | | | |
| AQ-Total | F | Y | 22.27 | ± 6.16 (49) | **2.26 (83)** | **0.03** |
|  |  | N | 19.97 | ± 6.41 (158) |  |  |
|  | M | Y | 20.70 | ± 6.87 (54) | 1.72 (100) | 0.09 |
|  |  | N | 18.77 | ± 5.98 (90) |  |  |
|  | B | Y | 21.45 | ± 6.56 (103) | **2.52 (183)** | **0.01** |
|  |  | N | 19.53 | ± 6.27 (248) |  |  |
| SPQ-Total | F | Y | 95.53 | ± 16.66 (49) | 0.27 (78) | 0.79 |
|  |  | N | 94.79 | ± 16.10 (158) |  |  |
|  | M | Y | 91.17 | ± 16.33 (54) | 0.74 (114) | 0.46 |
|  |  | N | 89.07 | ± 16.77 (90) |  |  |
|  | B | Y | 93.24 | ± 16.56 (103) | 0.27 (191) | 0.79 |
|  |  | N | 92.71 | ± 16.55 (248) |  |  |
| SPQ-CogPer | F | Y | 38.67 | ± 8.89 (49) | -0.50 (75) | 0.65 |
|  |  | N | 39.33 | ± 8.21 (158) |  |  |
|  | M | Y | 34.52 | ± 8.69 (54) | -0.90 (115) | 0.37 |
|  |  | N | 35.88 | ± 9.01 (90) |  |  |
|  | B | Y | 36.50 | ± 8.99 (103) | -1.52 (184) | 0.13 |
|  |  | N | 38.08 | ± 8.65 (248) |  |  |
| **Customization** | | | | | | |
| AQ-Total | F | Y | 21.90 | ± 6.55 (58) | **1.92 (100)** | 0.058 |
|  |  | N | 19.97 | ± 6.30 (149) |  |  |
|  | M | Y | 20.96 | ± 5.86 (55) | **2.26 (124)** | **0.03** |
|  |  | N | 18.58 | ± 6.54 (89) |  |  |
|  | B | Y | 21.44 | ± 6.21 (113) | **2.77 (227)** | **0.006** |
|  |  | N | 19.45 | ± 6.41 (238) |  |  |
| SPQ-Total | F | Y | 93.93 | ± 17.31 (58) | -0.55 (96) | 0.58 |
|  |  | N | 95.37 | ± 15.79 (149) |  |  |
|  | M | Y | 93.36 | ± 15.03 (55) | 1.42 (108) | 0.16 |
|  |  | N | 87.69 | ± 17.20 (89) |  |  |
|  | B | Y | 93.66 | ± 16.17 (113) | 0.62 (227) | 0.54 |
|  |  | N | 92.50 | ± 16.72 (238) |  |  |
| SPQ-CogPer | F | Y | 38.21 | ± 8.66 (58) | -1.02 (99) | 0.31 |
|  |  | N | 39.55 | ± 8.24 (149) |  |  |
|  | M | Y | 36.73 | ± 9.27 (55) | 1.42 (108) | 0.16 |
|  |  | N | 34.53 | ± 8.59 (89) |  |  |
|  | B | Y | 37.49 | ± 8.95 (113) | -0.18 (215) | 0.85 |
|  |  | N | 37.67 | ± 8.70 (238) |  |  |

**Supplementary Table 9**: Multiple regressions of Video Game Motivations in relation to AQ and SPQ subscales

| **Motivations** | | | | | |
| --- | --- | --- | --- | --- | --- |
|  | Estimate | Std.Error | z value | Pr(>\|z\|) | Significance |
| **Social Interaction** |  |  |  |  |  |
| #(Intercept) | -1.46381 | 0.60872 | -2.405 | 0.01618 | * |
| #social | -0.17447 | 0.05944 | -2.935 | 0.00334 | ** |
| #AQ- Detail | 0.1348 | 0.05495 | 2.453 | 0.01416 | * |
| #SPQ- Anxiety | 0.0934 | 0.03567 | 2.618 | 0.00884 | ** |
| #SPQ- Magic | -0.0974 | 0.03344 | -2.913 | 0.00358 | ** |
| #sexM | 0.96596 | 0.2471 | 3.909 | 9.26E-05 | *** |
|  |  |  |  |  |  |
| **Stress Relief** | | | | | |
| #(Intercept) | -0.72755 | 0.45867 | -1.586 | 0.11269 |  |
| #social | 0.13741 | 0.04945 | 2.779 | 0.00546 | ** |
| #SPQ- Percep | 0.07582 | 0.03783 | 2.004 | 0.04503 | * |
| #sexM | 0.53779 | 0.24303 | 2.213 | 0.02691 | * |
|  |  |  |  |  |  |
| **Skill Development** | | | | | |
| #(Intercept) | -4.64888 | 0.83445 | -5.571 | 2.53E-08 | *** |
| #AQ- Switch | -0.13525 | 0.08319 | -1.626 | 0.10399 |  |
| #AQ- Detail | 0.20253 | 0.06588 | 3.074 | 0.00211 | ** |
| #SPQ- Ideas | 0.06224 | 0.03191 | 1.951 | 0.0511 |  |
| #SPQ- Anxiety | 0.09324 | 0.04058 | 2.297 | 0.02159 | * |
| #sexM | 1.38235 | 0.28588 | 4.836 | 1.33E-06 | *** |
|  |  |  |  |  |  |
| **Adrenaline Rush** | | | | | |
| #(Intercept) | -1.16298 | 0.51493 | -2.259 | 0.023914 | * |
| #AQ- Switch | -0.16016 | 0.07058 | -2.269 | 0.02325 | * |
| #AQ- Detail | 0.11838 | 0.05695 | 2.079 | 0.037647 | * |
| #AQ- Comm | 0.16872 | 0.06234 | 2.706 | 0.0068 | ** |
| #AQ- Imag | -0.1774 | 0.08315 | -2.133 | 0.032888 | * |
| #sexM | 0.89758 | 0.24615 | 3.646 | 0.000266 | *** |
|  |  |  |  |  |  |
| **Escape** | | | | | |
| #(Intercept) | 0.11754 | 0.52936 | 0.222 | 0.8243 |  |
| #SPQ- Constrict | -0.04482 | 0.0244 | -1.837 | 0.0663 |  |
| #SPQ- Eccentric | 0.13206 | 0.03339 | 3.955 | 7.65E-05 | *** |
| #SPQ- Magic | -0.05827 | 0.03092 | -1.885 | 0.0595 |  |
| #sexM | 0.182 | 0.2356 | 0.773 | 0.4398 |  |
|  |  |  |  |  |  |
| **Fantasy** | | | | | |
| #(Intercept) | -1.48079 | 0.50749 | -2.918 | 0.00352 | ** |
| #social | 0.10662 | 0.05079 | 2.099 | 0.03579 | * |
| #SPQ- Eccentric | 0.05627 | 0.0343 | 1.64 | 0.10095 |  |
| #SPQ- Magic | -0.07812 | 0.03523 | -2.217 | 0.02661 | * |
| #sexM | 0.57983 | 0.25422 | 2.281 | 0.02256 | * |
|  |  |  |  |  |  |
| **Customization** | | | | | |
| #(Intercept) | -1.99995 | 0.40449 | -4.944 | 7.64E-07 | *** |
| #AQ- Detail | 0.11274 | 0.05411 | 2.084 | 0.0372 | * |
| #AQ- Comm | 0.12304 | 0.05286 | 2.328 | 0.0199 | * |
| #sexM | 0.50972 | 0.23537 | 2.166 | 0.0303 | * |
|  |  |  |  |  |  |
| AQ-Total, SPQ-Total, SPQ-CogPer and Video Game Motivations | | | | | |
| **Social Interaction** | | | | | |
| #(Intercept) | -1.19859 | 0.51878 | -2.31 | 0.0209 | * |
| #AQ- total | 0.01578 | 0.02387 | 0.661 | 0.5084 |  |
| #sexM | 1.68097 | 0.75021 | 2.241 | 0.025 | * |
| #AQ- total:sexM | -0.0348 | 0.03558 | -0.978 | 0.3279 |  |
| **Stress Relief** | | | | | |
| #(Intercept) | -0.67564 | 0.49225 | -1.373 | 0.1699 |  |
| #AQ- total | 0.06044 | 0.02365 | 2.556 | 0.0106 | * |
| #sexM | 1.23751 | 0.77008 | 1.607 | 0.1081 |  |
| #AQ- total:sexM | -0.04006 | 0.03772 | -1.062 | 0.2882 |  |
|  |  |  |  |  |  |
| **Skill Development** | | | | | |
| #(Intercept) | -2.696705 | 0.683938 | -3.943 | 8.05E-05 | *** |
| #AQ- total | 0.048813 | 0.030057 | 1.624 | 0.104 |  |
| #sexM | 1.159698 | 0.90512 | 1.281 | 0.2 |  |
| #AQ- total:sexM | -0.003008 | 0.041232 | -0.073 | 0.942 |  |
|  |  |  |  |  |  |
| **Adrenaline Rush** | | | | | |
| #(Intercept) | -1.99021 | 0.58557 | -3.399 | 0.000677 | *** |
| #AQ- total | 0.03527 | 0.02633 | 1.339 | 0.18049 |  |
| #sexM | 2.07825 | 0.80111 | 2.594 | 0.009481 | ** |
| #AQ- total:sexM | -0.06012 | 0.03766 | -1.596 | 0.110383 |  |
|  |  |  |  |  |  |
| **Escape** | | | | | |
| #(Intercept) | -0.18713 | 0.4712 | -0.397 | 0.691 |  |
| #AQ- total | 0.02101 | 0.02204 | 0.953 | 0.341 |  |
| #sexM | 0.61061 | 0.72888 | 0.838 | 0.402 |  |
| #AQ- total:sexM | -0.01499 | 0.03501 | -0.428 | 0.668 |  |
|  |  |  |  |  |  |
| **Fantasy** | | | | | |
| #(Intercept) | -2.373863 | 0.592879 | -4.004 | 6.23E-05 | *** |
| #AQ- total | 0.056982 | 0.026244 | 2.171 | 0.0299 | * |
| #sexM | 0.897916 | 0.831424 | 1.08 | 0.2802 |  |
| #AQ- total:sexM | -0.008086 | 0.038263 | -0.211 | 0.8326 |  |
|  |  |  |  |  |  |
| **Customization** | | | | | |
| #(Intercept) | -1.93899 | 0.54933 | -3.53 | 0.000416 | *** |
| #AQ- total | 0.04756 | 0.02468 | 1.927 | 0.053986 |  |
| #sexM | 0.25863 | 0.80655 | 0.321 | 0.748468 |  |
| #AQ- total:sexM | 0.01306 | 0.03742 | 0.349 | 0.727016 |  |
|  |  |  |  |  |  |
| **Social Interaction** | | | | | |
| # | Estimate | Std. Error | z value | Pr(>\|z\|) |  |
| #(Intercept) | -0.982868 | 0.734704 | -1.338 | 0.181 |  |
| #SPQ- CogPer | 0.002809 | 0.018309 | 0.153 | 0.878 |  |
| #sexM | 1.547661 | 1.008401 | 1.535 | 0.125 |  |
| #SPQ- CogPer:sexM | -0.015623 | 0.026328 | -0.593 | 0.553 |  |
|  |  |  |  |  |  |
|  |  |  |  |  |  |
| **Stress Relief** | | | | | |
| # | Estimate | Std. Error | z value | Pr(>\|z\|) |  |
| #(Intercept) | 0.413305 | 0.690974 | 0.598 | 0.55 |  |
| #SPQ- CogPer | 0.003351 | 0.017277 | 0.194 | 0.846 |  |
| #sexM | -1.066683 | 1.034617 | -1.031 | 0.303 |  |
| #SPQ- CogPer:sexM | 0.043189 | 0.02804 | 1.54 | 0.123 |  |
|  |  |  |  |  |  |
| **Skill Development** | | | | | |
| #(Intercept) | -2.4647 | 0.95275 | -2.587 | 0.00968 | ** |
| #SPQ- CogPer | 0.02023 | 0.02327 | 0.869 | 0.38462 |  |
| #sexM | 0.47192 | 1.21742 | 0.388 | 0.69828 |  |
| #SPQ- CogPer:sexM | 0.01779 | 0.0309 | 0.576 | 0.56489 |  |
|  |  |  |  |  |  |
| **Adrenaline Rush** | | | | | |
| #(Intercept) | -1.426867 | 0.808792 | -1.764 | 0.0777 |  |
| #SPQ- CogPer | 0.004435 | 0.020112 | 0.22 | 0.8255 |  |
| #sexM | 0.434307 | 1.074938 | 0.404 | 0.6862 |  |
| #SPQ- CogPer:sexM | 0.012431 | 0.02787 | 0.446 | 0.6556 |  |
|  |  |  |  |  |  |
| **Escape** | | | | | |
| #(Intercept) | 1.37239 | 0.69288 | 1.981 | 0.0476 | * |
| #SPQ- CogPer | -0.02874 | 0.0172 | -1.671 | 0.0947 |  |
| #sexM | -1.77272 | 0.99445 | -1.783 | 0.0747 |  |
| #SPQ- CogPer:sexM | 0.05556 | 0.02628 | 2.114 | 0.0345 | * |
|  |  |  |  |  |  |
| **Fantasy** | | | | | |
| # | Estimate | Std. Error | z value | Pr(>\|z\|) |  |
| #(Intercept) | -0.804764 | 0.775779 | -1.037 | 0.3 |  |
| #SPQ- CogPer | -0.009384 | 0.019529 | -0.481 | 0.631 |  |
| #sexM | 0.907813 | 1.050212 | 0.864 | 0.387 |  |
| #SPQ- CogPer:sexM | -0.008057 | 0.027669 | -0.291 | 0.771 |  |
|  |  |  |  |  |  |
| **Customization** | | | | | |
| #(Intercept) | -0.1958 | 0.7316 | -0.268 | 0.789 |  |
| #SPQ- CogPer | -0.01923 | 0.01852 | -1.038 | 0.2992 |  |
| #sexM | -1.29476 | 1.03134 | -1.255 | 0.2093 |  |
| #SPQ- CogPer:sexM | 0.04756 | 0.02703 | 1.76 | 0.0785 |  |
|  |  |  |  |  |  |
| **Social Interaction** | | | | | |
| #(Intercept) | -1.213661 | 0.917 | -1.324 | 0.186 |  |
| #SPQ- total | 0.003583 | 0.009483 | 0.378 | 0.706 |  |
| #sexM | 1.443336 | 1.301267 | 1.109 | 0.267 |  |
| #SPQ- total:sexM | -0.004901 | 0.013857 | -0.354 | 0.724 |  |
|  |  |  |  |  |  |
| **Stress Relief** | | | | | |
| # | Estimate | Std. Error | z value | Pr(>\|z\|) |  |
| #(Intercept) | -0.642217 | 0.858115 | -0.748 | 0.454 |  |
| #SPQ- total | 0.012551 | 0.008983 | 1.397 | 0.162 |  |
| #sexM | -0.262733 | 1.335065 | -0.197 | 0.844 |  |
| #SPQ- total:sexM | 0.008444 | 0.01459 | 0.579 | 0.563 |  |
|  |  |  |  |  |  |
| **Skill Development** | | | | | |
| #(Intercept) | -3.138954 | 1.208632 | -2.597 | 0.0094 | ** |
| #SPQ- total | 0.01533 | 0.012227 | 1.254 | 0.2099 |  |
| #sexM | 0.920959 | 1.578383 | 0.583 | 0.5596 |  |
| #SPQ- total:sexM | 0.002185 | 0.016411 | 0.133 | 0.8941 |  |
|  |  |  |  |  |  |
| **Adrenaline Rush** | | | | | |
| #(Intercept) | -1.67989 | 1.011333 | -1.661 | 0.0967 |  |
| #SPQ- total | 0.004482 | 0.010431 | 0.43 | 0.6674 |  |
| #sexM | 0.986395 | 1.382736 | 0.713 | 0.4756 |  |
| #SPQ- total:sexM | -0.001151 | 0.014661 | -0.079 | 0.9374 |  |
|  |  |  |  |  |  |
| **Escape** | | | | | |
| #(Intercept) | 0.500473 | 0.836873 | 0.598 | 0.5498 |  |
| #SPQ- total | -0.002713 | 0.00868 | -0.313 | 0.7546 |  |
| #sexM | -1.804932 | 1.279633 | -1.411 | 0.1584 |  |
| #SPQ- total:sexM | 0.023409 | 0.013824 | 1.693 | 0.0904 |  |
|  |  |  |  |  |  |
| **Fantasy** | | | | | |
| #(Intercept) | -1.44145 | 0.983138 | -1.466 | 0.143 |  |
| #SPQ- total | 0.002844 | 0.010166 | 0.28 | 0.78 |  |
| #sexM | 0.23317 | 1.377424 | 0.169 | 0.866 |  |
| #SPQ- total:sexM | 0.004895 | 0.014616 | 0.335 | 0.738 |  |
|  |  |  |  |  |  |
| **Customization** | | | | | |
| #(Intercept) | -0.424867 | 0.913075 | -0.465 | 0.642 |  |
| #SPQ- total | -0.005479 | 0.009538 | -0.574 | 0.566 |  |
| #sexM | -2.002969 | 1.358895 | -1.474 | 0.14 |  |
| #SPQ- total:sexM | 0.026974 | 0.014456 | 1.866 | 0.062 |  |

* < denotes significance at <0.05

** < denotes significance at < 0.01

*** denotes significance at < 0.001

**Supplementary Table 10**: Pearson Correlations for Reaction Times and Targeting Times

in relation to AQ and SPQ scales. P values under 0.05 or near are shown in brackets.

|  | | |
| --- | --- | --- |
|  | **Reaction Time (ms)** | **Targeting Time (ms)** |
| **Females** | **N = 144** | **N = 195** |
| AQ: Social | -0.16 [0.02] | 0.19 [0.006] |
| AQ- Switch | -0.09 | 0.06 |
| AQ- Detail | -0.13 [0.06] | 0.04 |
| AQ- Comm | -0.10 | 0.09 |
| AQ- Imag | -0.00 | -0.02 |
| AQ: Total | -0.17 [0.01] | 0.13 [0.06] |
| SPQ- Ideas | -0.08 | -0.05 |
| SPQ- Constrict | -0.07 | -0.04 |
| SPQ- Eccentric | -0.20 [0.0042] | 0.09 |
| SPQ- Anxiety | -0.16 [0.02] | 0.14 [0.05] |
| SPQ- Magic | -0.07 | -0.14 [0.06] |
| SPQ- Speech | -0.03 | -0.05 |
| SPQ- Percep | -0.05 | -0.09 |
| SPQ- Interpersonal | -0.13 | 0.05 |
| Disorganized | -0.15 [0.03] | 0.03 |
| SPQ- Cog-Per | -0.10 | -0.12 |
| SPQ- Total | -0.16 [0.02] | -0.03 |
| **Males** | **N = 144** | **N = 140** |
| AQ- Social | -0.01 | 0.04 |
| AQ- Switch | 0.02 | -0.03 |
| AQ- Detail | 0.05 | 0.08 |
| AQ- Comm | -0.02 | 0.04 |
| AQ- Imag | -0.05 | -0.02 |
| AQ- Total | 0.00 | 0.04 |
| SPQ- Ideas | -0.04 | -0.06 |
| SPQ- Constrict | -0.10 | 0.15 |
| SPQ- Eccentric | 0.04 | -0.01 |
| SPQ- Anxiety | -0.09 | 0.13 |
| SPQ- Magic | 0.32 [8.86E-05] | -0.29 [0.001] |
| SPQ- Speech | -0.02 | 0.07 |
| SPQ- Percep | 0.07 | -0.12 |
| SPQ- Interpersonal | -0.12 | 0.17 [0.05] |
| SPQ- Disorganized | 0.01 | 0.03 |
| SPQ- Cog- Per | 0.13 | -0.20 [0.03] |
| SPQ- Total | 0.02 | -0.02 |
| **Both Sexes** | **N = 348** | **N = 335** |
| AQ- Social | -0.07 | 0.09 |
| AQ- Switch | -0.03 | 0.00 |
| AQ- Detail | -0.05 | 0.03 |
| AQ- Comm | -0.06 | 0.06 |
| AQ- Imag | -0.02 | -0.02 |
| AQ- Total | -0.08 | 0.06 |
| SPQ- Ideas | -0.03 | -0.09 |
| SPQ- Constrict | -0.10 | 0.06 |
| SPQ- Eccentric | -0.11 [0.04] | 0.06 |
| SPQ- Anxiety | -0.08 | 0.07 |
| SPQ- Magic | 0.14 [0.01] | -0.26 [1.17E-06] |
| SPQ- Speech | -0.00 | -0.04 |
| SPQ- Percep | 0.02 | -0.13 [0.02] |
| SPQ- Interpersonal | -0.11 [0.04] | 0.08 |
| SPQ- Disorganized | -0.07 | 0.02 |
| SPQ- Cog-Percep | 0.05 | -0.21 [0.00013] |
| SPQ- Total | -0.05 | -0.07 |

**Supplementary Table 11.** Multiple regressions of Reaction Times and Targeting Times in relation to AQ and SPQ subscales

| **Correlation of Targeting time to Reaction Time** | | | | | |
| --- | --- | --- | --- | --- | --- |
| # | Estimate | Std. Error | t value | Pr(>\|t\|) | Significance |
| #(Intercept) | 79.7729 | 58.5319 | 1.363 | 0.1738 |  |
| #rt | -0.847 | 0.2028 | -4.176 | 3.80E-05 | *** |
| #sexM | 246.6696 | 87.0867 | 2.832 | 0.0049 | ** |
| #rt:sexM | -0.6986 | 0.3107 | -2.249 | 0.0252 | * |
|  |  |  |  |  |  |
| **Multiple regression ** of Reaction times in relation to AQ and SPQ subscales** | | | | | |
| # | Estimate | Std. Error | t value | Pr(>\|t\|) | Significance |
| #(Intercept) | 302.89688 | 13.19073 | 22.963 | < 2.00E-16 | *** |
| #AQ - Social | -0.39209 | 1.12491 | -0.349 | 0.727644 |  |
| #AQ- Switch | 0.59866 | 1.14814 | 0.521 | 0.602425 |  |
| #AQ- Detail | -1.08063 | 0.84608 | -1.277 | 0.202412 |  |
| #AQ- Comm | 0.39686 | 1.19042 | 0.333 | 0.73906 |  |
| #AQ- Imag | -0.22032 | 1.24019 | -0.178 | 0.859104 |  |
| #SPQ- Ideas | -0.25072 | 0.51144 | -0.49 | 0.624304 |  |
| #SPQ- Constrict | -0.15577 | 0.44731 | -0.348 | 0.727879 |  |
| #SPQ- Eccentric | -0.68387 | 0.604 | -1.132 | 0.258347 |  |
| #SPQ- Anxiety | -0.79811 | 0.62891 | -1.269 | 0.20531 |  |
| #SPQ- Magic | 0.91986 | 0.55541 | 1.656 | 0.098622 |  |
| #SPQ- Speech | 0.02644 | 0.67003 | 0.039 | 0.968543 |  |
| #SPQ- Percep | 0.3285 | 0.70156 | 0.468 | 0.639913 |  |
| #sexM | -14.48138 | 4.07952 | -3.55 | 0.000441 | *** |
|  |  |  |  |  |  |
| **Multiple Regression of Targeting times in relation to AQ and SPQ subscales** | | | | | |
| # | Estimate | Std. Error | t value | Pr(>\|t\|) | Significance |
| #(Intercept) | -145.9983 | 39.9941 | -3.65 | 0.000305 | *** |
| #social | 2.8928 | 3.4525 | 0.838 | 0.402711 |  |
| #AQ- Switch | -2.6727 | 3.4656 | -0.771 | 0.44114 |  |
| #AQ- Detail | 4.5847 | 2.607 | 1.759 | 0.079592 |  |
| #AQ- Comm | 0.1444 | 3.6509 | 0.04 | 0.968478 |  |
| #AQ- Imag | -2.717 | 3.8086 | -0.713 | 0.476125 |  |
| #SPQ- Ideas | -1.1812 | 1.5423 | -0.766 | 0.44433 |  |
| #SPQ- Constrict | -0.3094 | 1.3722 | -0.225 | 0.821736 |  |
| #SPQ- Eccentric | 1.018 | 1.8482 | 0.551 | 0.582138 |  |
| #SPQ- Anxiety | 3.3686 | 1.9045 | 1.769 | 0.077888 |  |
| #SPQ- Magic | -4.6986 | 1.6803 | -2.796 | 0.005481 | ** |
| #SPQ- Speech | 0.7816 | 2.0492 | 0.381 | 0.703152 |  |
| #SPQ- Percep | -2.5869 | 2.1137 | -1.224 | 0.221887 |  |
| #sexM | 65.4571 | 12.452 | 5.257 | 2.68E-07 | *** |
|  |  |  |  |  |  |

* < denotes significance at <0.05

** < denotes significance at < 0.01

*** denotes significance at < 0.001

**Supplementary Table 12**: Pearson Correlations of Video Game Usage in relation to Reaction Times (RT) and Targeting Times (RT); r = Pearson correlation. N=195 (females), 140 (males), 335 (both sexes).

| **Video Game Usage Variable** | | | | | | | | | | |
| --- | --- | --- | --- | --- | --- | --- | --- | --- | --- | --- |
|  | **Weekday (h)** | | **Weekend (h)** | | **Self report frequency** | | **Self report usage** | | **Self report spare time** | |
|  | **r** | **p** | **r** | **p** | **r** | **p** | **r** | **p** | **r** | **p** |
| **Females** | | | | | | | | | | |
| RT | -0.06 | 0.38 | -0.11 | 0.11 | -0.18 | 6.16E-04 | 0.11 | 0.11 | -0.09 | 0.22 |
| TT | 0.33 | 2.11E-06 | 0.34 | 1.30E-06 | 0.29 | 4.32E-05 | -0.21 | 0.004 | 0.32 | 5.2E-06 |
| **Males** | | | | | | | | | | |
| RT | -0.23 | 0.006 | -0.21 | 0.01 | -0.23 | 0.005 | 0.15 | 0.07 | -0.20 | 0.01 |
| TT | 0.29 | 0.0005 | 0.30 | 0.0003 | 0.26 | 0.002 | -0.29 | 0.0004 | 0.27 | 0.001 |
| **Both Sexes** | | | | | | | | | | |
| RT | -0.21 | 6.19E-05 | -0.23 | 1.18E-05 | 0.19 | 4.16E-04 | 0.19 | 4.16E-04 | -0.21 | 5.58E-05 |
| TT | 0.39 | 8.86E-14 | 0.41 | 5.55E-15 | 0.38 | 1.06E-12 | -0.32 | 1.29E-09 | 0.39 | 2.28E-13 |
|  |  |  |  |  |  |  |  |  |  |  |
|  |  |  |  |  |  |  |  |  |  |  |

**Supplementary Table 13**. Stepwise regression analyses for AQ and SPQ subscales in relation to Video Game Usage Variables

| **Usage Self Report to AQ/SPQ and Reaction time and Targeting Time** | | | | | |
| --- | --- | --- | --- | --- | --- |
| # | Estimate | Std. Error | t value | Pr(>\|t\|) | Significance |
| #(Intercept) | 3.7781795 | 0.310519 | 12.167 | < 2.00E-16 | *** |
| #AQ- Detail | -0.0647273 | 0.0329048 | -1.967 | 0.05 | * |
| #SPQ- Eccentric | -0.0410315 | 0.0190243 | -2.157 | 0.0317 | * |
| #sexM | -0.8282184 | 0.1541396 | -5.373 | 1.47E-07 | *** |
| #targett | -0.0030079 | 0.0006893 | -4.363 | 1.72E-05 | *** |
| #Residual standard error: 1.315 on 328 degrees of freedom | | | | |  |
| #Multiple R-squared: 0.1971, | Adjusted R-squared: 0.1873 | | |  |  |
| #F-statistic: 20.13 on 4 and 328 DF, p-value: 7.714e-15 | | | | |  |
|  |  |  |  |  |  |
| **Frequency Self Report to AQ/SPQ and Reaction Time and Targeting Time** | | | | | |
| #(Intercept) | 4.4749143 | 0.4736514 | 9.448 | < 2.00E-16 | *** |
| #social | 0.0670621 | 0.0430508 | 1.558 | 0.1203 |  |
| #SPQ- Ideas | -0.0396469 | 0.0232306 | -1.707 | 0.0888 |  |
| #SPQ- Eccentric | 0.0463542 | 0.0293338 | 1.58 | 0.115 |  |
| #sexM | 1.8339238 | 0.2169792 | 8.452 | 9.58E-16 | *** |
| #targett | 0.0044864 | 0.0009647 | 4.65 | 4.81E-06 | *** |
| #Residual standard error: 1.824 on 327 degrees of freedom | | | | |  |
| #Multiple R-squared: 0.313, | Adjusted R-squared: 0.3025 | | |  |  |
| #F-statistic: 29.8 on 5 and 327 DF, p-value: < 2.2e-16 | | | | |  |
|  |  |  |  |  |  |
| **Weekday Time (h) to AQ/SPQ and Reaction Time and Targeting Time** | | | | | |
| #(Intercept) | 0.9345684 | 0.5162202 | 1.81 | 0.0712 |  |
| #AQ- Detail | 0.0673087 | 0.0353225 | 1.906 | 0.0576 |  |
| #SPQ- Constrict | -0.0296439 | 0.0169008 | -1.754 | 0.0804 |  |
| #SPQ- Anxiety | 0.0319666 | 0.0209799 | 1.524 | 0.1286 |  |
| #SPQ- Speech | 0.0522258 | 0.0255035 | 2.048 | 0.0414 | * |
| #SPQ- Percep | -0.043023 | 0.026713 | -1.611 | 0.1082 |  |
| #sexM | 1.1057321 | 0.1713112 | 6.455 | 3.95E-10 | *** |
| #targett | 0.0040805 | 0.0007478 | 5.457 | 9.64E-08 | *** |
| #Residual standard error: 1.403 on 325 degrees of freedom | | | | |  |
| #Multiple R-squared: 0.2645, | Adjusted R-squared: 0.2486 | | |  |  |
| #F-statistic: 16.69 on 7 and 325 DF, p-value: < 2.2e-16 | | | | |  |
|  |  |  |  |  |  |
| **Weekend Time (h) to AQ/SPQ and Reaction Time and Targeting Time** | | | | | |
| #(Intercept) | 1.271434 | 0.556319 | 2.285 | 0.0229 | * |
| #AQ- Comm | 0.097316 | 0.051329 | 1.896 | 0.0589 |  |
| #SPQ- Speech | 0.075818 | 0.035472 | 2.137 | 0.0333 | * |
| #sexM | 1.745328 | 0.238275 | 7.325 | 1.87E-12 | *** |
| #targett | 0.006407 | 0.001061 | 6.037 | 4.24E-09 | *** |
| #Residual standard error: 2.025 on 328 degrees of freedom | | | | |  |
| #Multiple R-squared: 0.2948, | Adjusted R-squared: 0.2s862 | | |  |  |
| #F-statistic: 34.27 on 4 and 328 DF, p-value: < 2.2e-16 | | | | |  |
|  |  |  |  |  |  |
| **Spare Time Self Report to AQ/SPQ and Reaction Time and Targeting Time** | | | | | |
| # | Estimate | Std. Error | t value | Pr(>\|t\|) |  |
| #(Intercept) | 2.1384391 | 0.2301692 | 9.291 | < 2.00E-16 | *** |
| #SPQ- Eccentric | 0.0470214 | 0.0149182 | 3.152 | 0.00177 | ** |
| #SPQ- Magic | -0.0353157 | 0.0157008 | -2.249 | 0.02516 | * |
| #sexM | 0.7533648 | 0.1222097 | 6.165 | 2.07E-09 | *** |
| #targett | 0.0027803 | 0.0005501 | 5.055 | 7.18E-07 | *** |
| #Residual standard error: 1.028 on 328 degrees of freedom | | | | |  |
| #Multiple R-squared: 0.2811, | Adjusted R-squared: 0.2723 | | |  |  |
| #F-statistic: 32.06 on 4 and 328 DF, p-value: < 2.2e-16 | | | | |  |

* < denotes significance at <0.05

** < denotes significance at < 0.01

*** denotes significance at < 0.001

**Supplementary Table 14**: ANCOVAs of Reaction Time and Video Game Usage in relation to Video Game Usage variables

| **Reaction Time and Video Game Usage on Video Game Usage Variables** | | | | | |
| --- | --- | --- | --- | --- | --- |
| # | Estimate | Std. Error | t value | Pr(>\|t\|) | Significance |
| **Usage Self Report** | | | | | |
| #(Intercept) | 1.968349 | 0.620888 | 3.17 | 0.00166 | ** |
| #rt | 0.005145 | 0.002143 | 2.401 | 0.01688 | * |
| #sexM | -0.920412 | 0.152408 | -6.039 | 4.01E-09 | *** |
|  |  |  |  |  |  |
| **Frequency Self Report** | | | | | |
| #(Intercept) | 5.092265 | 0.857797 | 5.936 | 7.10E-09 | *** |
| #rt | -0.005014 | 0.00296 | -1.694 | 0.0912 | . |
| #sexM | 2.199312 | 0.210561 | 10.445 | < 2.00E-16 | *** |
|  |  |  |  |  |  |
| **Weekday time (h)** | | | | | |
| #(Intercept) | 2.590949 | 0.665821 | 3.891 | 0.00012 | *** |
| #rt | -0.006174 | 0.002298 | -2.687 | 0.00756 | ** |
| #sexM | 1.251552 | 0.163437 | 7.658 | 1.92E-13 | *** |
|  |  |  |  |  |  |
| **Weekend time (h)** | | | | | |
| #(Intercept) | 4.388712 | 0.956443 | 4.589 | 6.26E-06 | *** |
| #rt | -0.009851 | 0.003301 | -2.984 | 0.00304 | ** |
| #sexM | 2.035239 | 0.234776 | 8.669 | < 2.00E-16 | *** |
|  |  |  |  |  |  |
| **Self Report Spare Time** | | | | | |
| #(Intercept) | 3.092605 | 0.486386 | 6.358 | 6.47E-10 | *** |
| #rt | -0.004399 | 0.001679 | -2.62 | 0.00917 | ** |
| #sexM | 1.004191 | 0.119392 | 8.411 | 1.09E-15 | *** |
|  |  |  |  |  |  |
| **ANCOVA of Targeting Time and Video Game Usage on Video Game Usage Variables** | | | | | |
| **Self Report Frequency** | | | | | |
| #(Intercept) | 4.5429709 | 0.2048749 | 22.174 | < 2.00E-16 | *** |
| #targett | 0.0048992 | 0.0009614 | 5.096 | 5.84E-07 | *** |
| #sexM | 1.8295254 | 0.2145574 | 8.527 | 5.39E-16 | *** |
|  |  |  |  |  |  |
| **Weekday Time (h)** | | | | | |
| #(Intercept) | 1.5788947 | 0.1577565 | 10.008 | < 2e-16 | *** |
| #targett | 0.0044279 | 0.0007403 | 5.981 | 5.73e-09 | *** |
| #sexM | 0.9709525 | 0.1652122 | 5.877 | 1.02e-08 | *** |
|  |  |  |  |  |  |
| **Weekend Time (h)** | | | | | |
| #(Intercept) | 2.679045 | 0.227408 | 11.781 | < 2.00E-16 | *** |
| #targett | 0.006582 | 0.001067 | 6.168 | 2.02E-09 | *** |
| #sexM | 1.672245 | 0.238156 | 7.022 | 1.24E-11 | *** |
|  |  |  |  |  |  |
| **Spare Time Self Report** | | | | | |
| #(Intercept) | 2.38157 | 0.116359 | 20.468 | < 2.00E-16 | *** |
| #targett | 0.003124 | 0.000546 | 5.722 | 2.36E-08 | *** |
| #sexM | 0.810637 | 0.121858 | 6.652 | 1.19E-10 | *** |
|  |  |  |  |  |  |
| **Usage Self Report** | | | | | |
| #(Intercept) | 2.9408571 | 0.1476548 | 19.92 | < 2.00E-16 |  |
| #targett | -0.0031597 | 0.0006929 | -4.56 | 7.20E-06 | *** |
| #sexM | -0.814947 | 0.154633 | -5.27 | 2.46E-07 | *** |

* < denotes significance at <0.05

** < denotes significance at < 0.01

*** denotes significance at < 0.001

**Supplementary Table 15**: Analysis of Reaction Time and Targeting Time in relation to Video Game Genre preferences

| **Variables** | | **Scores**  **Mean ± SD (N)** | | | **T (df)** | **p value** |
| --- | --- | --- | --- | --- | --- | --- |
| **Reaction time (ms)** | | | | | | |
| Action | F | Y | 282.93 | ± 34.32 (59) | -0.89 (106) | 0.38 |
|  |  | N | 287.64 | ± 33.82 (145) |  |  |
|  | M | Y | 268.00 | ± 31.45 (111) | -1.85 (43) | 0.07 |
|  |  | N | 283.01 | ± 43.31 (33) |  |  |
|  | B | Y | 273.19 | ± 33.15 (170) | **-3.68 (346)** | **2.55E-04** |
|  |  | N | 286.78 | ± 35.68 (178) |  |  |
| Platformer | F | Y | 282.39 | ± 31.10 (65) | -1.17 (140) | 0.24 |
|  |  | N | 288.09 | ± 35.16 (139) |  |  |
|  | M | Y | 273.96 | ± 31.97 (39) | 0.55 (76) | 0.58 |
|  |  | N | 270.51 | ± 36.07 (105) |  |  |
|  | B | Y | 279.23 | ± 31.54 (104) | -0.33 (224) | 0.74 |
|  |  | N | 280.53 | ± 36.54 (244) |  |  |
| Puzzle | F | Y | 284.19 | ± 35.91 (105) | -0.91 (201) | 0.36 |
|  |  | N | 288.49 | ± 31.76 (99) |  |  |
|  | M | Y | 269.56 | ± 32.79 (43) | -0.44 (86) | 0.66 |
|  |  | N | 272.24 | ± 35.94 (101) |  |  |
|  | B | Y | 279.94 | ± 35.55 (148) | -0.09 (313) | 0.93 |
|  |  | N | 280.29 | ± 34.82 (200) |  |  |
| RPG | F | Y | 277.29 | ± 23.28 (44) | **-2.54 (106)** | **0.01** |
|  |  | N | 288.75 | ± 36.00 (160) |  |  |
|  | M | Y | 265.63 | ± 34.55 (74) | **-2.08 (142)** | **0.04** |
|  |  | N | 277.59 | ± 34.52 (70) |  |  |
|  | B | Y | 269.97 | ± 31.24 (118) | **-4.13 (266)** | **0.00005** |
|  |  | N | 285.35 | ± 35.85 (230) |  |  |
| Strategy | F | Y | 276.22 | ± 28.82 (28) | -1.93 (40) | 0.06 |
|  |  | N | 287.88 | ± 34.49 (176) |  |  |
|  | M | Y | 266.79 | ± 32.26 (68) | -1.53 (142) | 0.13 |
|  |  | N | 275.60 | ± 36.87 (76) |  |  |
|  | B | Y | 269.54 | ± 31.45 (96) | **-3.74 (193)** | **0.0002** |
|  |  | N | 284.17 | ± 35.60 (252) |  |  |
| Sports | F | Y | 281.11 | ± 31.06 (29) | -0.95 (40) | 0.35 |
|  |  | N | 287.13 | ± 34.41 (175) |  |  |
|  | M | Y | 269.91 | ± 31.29 (44) | -0.37 (95) | 0.71 |
|  |  | N | 272.12 | ± 36.55 (100) |  |  |
|  | B | Y | 274.36 | ± 31.47 (73) | -1.71 (126) | 0.09 |
|  |  | N | 281.67 | ± 35.87 (275) |  |  |
| Racing | F | Y | 281.09 | ± 31.36 (43) | -1.19 (72) | 0.24 |
|  |  | N | 287.66 | ± 34.57 (161) |  |  |
|  | M | Y | 271.32 | ± 27.60 (32) | -0.03 (66) | 0.98 |
|  |  | N | 271.48 | ± 36.87 (112) |  |  |
|  | B | Y | 276.92 | ± 30.02 (75) | -1.00 (139) | 0.32 |
|  |  | N | 281.02 | ± 36.35 (273) |  |  |
| Construction | F | Y | 280.34 | ± 25.27 (64) | **-1.95 (172)** | **0.053** |
|  |  | N | 288.99 | ± 37.01 (140) |  |  |
|  | M | Y | 268.29 | ± 28.74 (57) | -0.93 (139) | 0.35 |
|  |  | N | 273.51 | ± 38.48 (87) |  |  |
|  | B | Y | 274.66 | ± 27.51 (121) | **-2.36 (316)** | **0.02** |
|  |  | N | 283.06 | ± 38.25 (227) |  |  |
| Social Simulation | F | Y | 287.22 | ± 37.06 (75) | 0.29 (138) | 0.77 |
|  |  | N | 285.73 | ± 32.14 (129) |  |  |
|  | M | Y | 267.78 | ± 31.44 (20) | -0.55 (27) | 0.59 |
|  |  | N | 272.03 | ± 35.55 (124) |  |  |
|  | B | Y | 283.13 | ± 36.67 (95) | 0.95 (160) | 0.35 |
|  |  | N | 279.02 | ± 34.47 (253) |  |  |
| Idle | F | Y | 277.91 | ± 30.91 (20) | -1.26 (24) | 0.22 |
|  |  | N | 287.19 | ± 34.22 (184) |  |  |
|  | M | Y | 256.62 | ± 20.81 (15) | **-2.66 (25)** | **0.01** |
|  |  | N | 273.17 | ± 35.88 (129) |  |  |
|  | B | Y | 268.78 | ± 28.75 (35) | **-2.40 (46)** | **0.02** |
|  |  | N | 281.41 | ± 35.53 (313) |  |  |
| Targeting Time (ms) | | | | | | |
| Action | F | Y | -137.79 | ± 93.43 (57) | **2.37 (113)** | **0.02** |
|  |  | N | -173.65 | ± 101.37 (138) |  |  |
|  | M | Y | -89.49 | ± 109.95 (109) | 0.78 (47) | 0.44 |
|  |  | N | -107.73 | ± 115.64(31) |  |  |
|  | B | Y | -106.08 | ± 106.79 (166) | **4.75 (333)** | **3.00E-06** |
|  |  | N | -161.56 | ± 106.88 (169) |  |  |
| Platformer | F | Y | -149.74 | ± 85.98 (62) | 1.38 (144) | 0.17 |
|  |  | N | -169.43 | ± 105.93 (133) |  |  |
|  | M | Y | -87.77 | ± 105.53 (39) | 0.39 (74) | 0.70 |
|  |  | N | -95.75 | ± 113.57 (101) |  |  |
|  | B | Y | -125.81 | ± 98.29 (101) | 0.96 (220) | **0.34** |
|  |  | N | -137.63 | ± 115.02 (234) |  |  |
| Puzzle | F | Y | -158.50 | ± 103.94 (101) | 0.68 (193) | 0.50 |
|  |  | N | -168.18 | ± 98.37 (94) |  |  |
|  | M | Y | -82.28 | ± 117.54 (42) | 0.76 (72) | 0.45 |
|  |  | N | -98.35 | ± 108.45 (98) |  |  |
|  | B | Y | -136.11 | ± 113.18 (143) | -0.29 (298) | 0.77 |
|  |  | N | -132.54 | ± 108.26 (192) |  |  |
| RPG | F | Y | -103.12 | ± 86.54 (43) | **5.01 (75)** | **3.57E-06** |
|  |  | N | -180.15 | ± 97.49 (152) |  |  |
|  | M | Y | -70.55 | ± 94.43 (71) | **2.52 (128)** | **0.01** |
|  |  | N | -117.17 | ± 122.15 (69) |  |  |
|  | B | Y | -82.84 | ± 92.52 (114) | **6.83 (264)** | **5.88E-11** |
|  |  | N | -160.49 | ± 109.50 (221) |  |  |
| Strategy | F | Y | -127.35 | ± 85.08 (26) | **2.24 (37)** | **0.03** |
|  |  | N | -168.68 | ± 101.45 (169) |  |  |
|  | M | Y | -72.66 | ± 107.46 (66) | **2.13 (137)** | **0.03** |
|  |  | N | -112.14 | ± 111.64 (74) |  |  |
|  | B | Y | -88.11 | ± 104.16 (92) | **4.92 (169)** | **2.02E-06** |
|  |  | N | -151.46 | ± 107.63 (243) |  |  |
| Sports | F | Y | -161.60 | ± 109.97 (26) | 0.08 (32) | 0.94 |
|  |  | N | -163.41 | ± 98.99 (169) |  |  |
|  | M | Y | -97.12 | ± 115.55 (44) | -0.25 (80) | 0.80 |
|  |  | N | -91.88 | ± 109.54 (96) |  |  |
|  | B | Y | -121.07 | ± 116.99 (70) | 1.06 (102) | 0.29 |
|  |  | N | -137.50 | ± 108.35 (265) |  |  |
| Racing | F | Y | -155.49 | ± 82.51 (43) | 0.65 (84) | 0.52 |
|  |  | N | -165.34 | ± 104.83 (152) |  |  |
|  | M | Y | -94.13 | ± 111.02 (31) | -0.03 (49) | 0.97 |
|  |  | N | -93.36 | ± 111.60 (109) |  |  |
|  | B | Y | -129.79 | ± 99.55 (74) | 0.41 (131) | 0.69 |
|  |  | N | -135.28 | ± 113.23 (261) |  |  |
| Construction | F | Y | -138.17 | ± 88.28 (62) | 2.55 (138) | 0.01 |
|  |  | N | - 174.82 | ± 103.59 (133) |  |  |
|  | M | Y | -71.91 | ± 103.27 (55) | **1.91 (124)** | **0.058** |
|  |  | N | -107.52 | ± 114.26 (85) |  |  |
|  | B | Y | -107.02 | ± 100.83 (117) | **3.45 (261)** | **6.51E-04** |
|  |  | N | -148.58 | ± 112.53 (218) |  |  |
| Social simulation | F | Y | -140.47 | ± 95.89 (74) | **2.54 (160)** | **0.01** |
|  |  | N | -177.05 | ± 100.66 (121) |  |  |
|  | M | Y | -47.98 | ± 99.34 (19) | 2.11 (26) | 0.04 |
|  |  | N | -100.68 | ± 111.49 (121) |  |  |
|  | B | Y | -121.58 | ± 103.11 (93) | 1.34 (181) | 0.18 |
|  |  | N | -138.86 | ± 112.69 (242) |  |  |
| Idle | F | Y | -108.09 | ± 84.93 (20) | **3.00 (25)** | **0.006** |
|  |  | N | -169.46 | ± 100.11 (175) |  |  |
|  | M | Y | -56.05 | ± 89.38 (15) | 1.67 (20) | 0.11 |
|  |  | N | -98.03 | ± 112.86 (125) |  |  |
|  | B | Y | -85.79 | ± 89.46 (35) | **3.28 (47)** | **0.002** |
|  |  | N | -139.70 | ± 111.17 (300) |  |  |

**Supplementary Table 16**: ANCOVAs of Reaction Time and Targeting Times in relation to Video Game genre preferences (analyzed without interaction terms)

| Reaction Time (ms) with Video Game Genres | | | | | |
| --- | --- | --- | --- | --- | --- |
|  | Estimate | Std. Error | t value | Pr(>\|t\|) | Significance |
| **Puzzle** | | | | | |
| #(Intercept) | 0.7251463 | 0.2195374 | 3.303 | 0.00106 | ** |
| #rt | -0.0007351 | 0.0007577 | -0.97 | 0.33263 |  |
| #sexM | -0.2269995 | 0.0538892 | -4.212 | 3.23E-05 | *** |
|  |  |  |  |  |  |
| **Action** | | | | | |
| #(Intercept) | 0.6949461 | 0.1991283 | 3.49 | 0.000546 | *** |
| #rt | -0.0014173 | 0.0006872 | -2.062 | 0.03993 | * |
| #sexM | 0.4605932 | 0.0488795 | 9.423 | 2.00E-16 | *** |
|  |  |  |  |  |  |
| **Platformer** | | | | | |
| #(Intercept) | 0.4269376 | 0.2080752 | 2.052 | 0.0409 | * |
| #rt | -0.0003783 | 0.0007181 | -0.527 | 0.5986 |  |
| #sexM | -0.0534066 | 0.0510757 | -1.046 | 0.2965 |  |
| **RPG** | | | | | |
| #(Intercept) | 0.7945085 | 0.2024794 | 3.924 | 0.000105 | *** |
| #rt | -0.0020219 | 0.0006988 | -2.893 | 0.004053 | ** |
| #sexM | 0.2682088 | 0.0497021 | 5.396 | 1.27E-07 | *** |
|  |  |  |  |  |  |
| **Strategy** | | | | | |
| #(Intercept) | 0.556261 | 0.187759 | 2.963 | 0.00326 | ** |
| #rt | -0.001464 | 0.000648 | -2.259 | 0.02453 | * |
| #sexM | 0.313255 | 0.046089 | 6.797 | 4.71E-11 | *** |
|  |  |  |  |  |  |
| **Sports** | | | | | |
| #(Intercept) | 0.2943877 | 0.1815429 | 1.622 | 0.105805 |  |
| #rt | -0.0005318 | 0.0006265 | -0.849 | 0.396625 |  |
| #sexM | 0.1555103 | 0.0445628 | 3.49 | 0.000546 | *** |
|  |  |  |  |  |  |
| **Racing** | | | | | |
| #(Intercept) | 0.3696849 | 0.1870099 | 1.977 | 0.0489 | * |
| #rt | -0.0005551 | 0.0006454 | -0.86 | 0.3904 |  |
| #sexM | 0.0032039 | 0.0459048 | 0.07 | 0.9444 |  |
|  |  |  |  |  |  |
| **Construction** | | | | | |
| #(Intercept) | 0.706017 | 0.215009 | 3.284 | 0.00113 | ** |
| #rt | -0.00137 | 0.000742 | -1.847 | 0.06565 | . |
| #sexM | 0.06178 | 0.052778 | 1.171 | 0.24258 |  |
|  |  |  |  |  |  |
| **Social Simulation** | | | | | |
| #(Intercept) | 3.70E-01 | 1.96E-01 | 1.883 | 0.0606 |  |
| #rt | -6.37E-06 | 6.77E-04 | -0.009 | 0.9925 |  |
| #sexM | -2.29E-01 | 4.82E-02 | -4.75 | 2.98E-06 | *** |
|  |  |  |  |  |  |
| **Idle** | | | | | |
| #(Intercept) | 0.371175 | 0.1361327 | 2.727 | 0.00673 | ** |
| #rt | -0.0009541 | 0.0004698 | -2.031 | 0.04305 | * |
| #sexM | -0.0080261 | 0.0334161 | -0.24 | 0.81033 |  |
|  |  |  |  |  |  |
| **Targeting Time (ms) with Video Game Genres** | | | |  |  |
| **Puzzle** | | | | | |
| #(Intercept) | 0.5599985 | 0.0539107 | 10.388 | < 2.00E-16 | *** |
| #targett | 0.0002577 | 0.000253 | 1.019 | 0.309 |  |
| #sexM | -0.2358953 | 0.0564586 | -4.178 | 3.76E-05 | *** |
|  |  |  |  |  |  |
| **Action** | | | | | |
| #(Intercept) | 0.3763286 | 0.0486922 | 7.729 | 1.30E-13 | *** |
| #targett | 0.0005149 | 0.0002285 | 2.254 | 0.0249 | * |
| #sexM | 0.4504042 | 0.0509934 | 8.833 | < 2.00E-16 | *** |
|  |  |  |  |  |  |
| **Platformer** | | | | | |
| #(Intercept) | 0.3650271 | 0.0511655 | 7.134 | 6.14E-12 | *** |
| #targett | 0.0002885 | 0.0002401 | 1.202 | 0.23 |  |
| #sexM | -0.05947 | 0.0535836 | -1.11 | 0.268 |  |
|  |  |  |  |  |  |
| **RPG** | | | | | |
| #(Intercept) | 0.4081728 | 0.0487543 | 8.372 | 1.61E-15 | *** |
| #targett | 0.0011501 | 0.0002288 | 5.027 | 8.17E-07 | *** |
| #sexM | 0.206538 | 0.0510585 | 4.045 | 6.51E-05 | *** |
|  |  |  |  |  |  |
| **Strategy** | | | | | |
| #(Intercept) | 0.2361688 | 0.0457106 | 5.167 | 4.12E-07 | *** |
| #targett | 0.0006302 | 0.0002145 | 2.938 | 0.00353 | ** |
| #sexM | 0.2942057 | 0.0478709 | 6.146 | 2.28E-09 | *** |
|  |  |  |  |  |  |
| **Sports** | | | | | |
| # | Estimate | Std. Error | t value | Pr(>\|t\|) |  |
| #(Intercept) | 1.28E-01 | 4.44E-02 | 2.888 | 0.00413 | ** |
| #targett | -3.21E-05 | 2.08E-04 | -0.154 | 0.8777 |  |
| #sexM | 1.83E-01 | 4.65E-02 | 3.943 | 9.81E-05 | *** |
|  |  |  |  |  |  |
| **Racing** | | | | | |
| #(Intercept) | 2.34E-01 | 4.64E-02 | 5.053 | 7.20E-07 | *** |
| #targett | 8.50E-05 | 2.18E-04 | 0.39 | 0.696 |  |
| #sexM | -5.00E-03 | 4.86E-02 | -0.103 | 0.918 |  |
|  |  |  |  |  |  |
| **Construction** | | | | | |
| #(Intercept) | 0.4399368 | 0.0524321 | 8.391 | 1.41E-15 | *** |
| #targett | 0.0007476 | 0.000246 | 3.039 | 0.00257 | ** |
| #sexM | 0.0228447 | 0.0549101 | 0.416 | 0.67765 |  |
|  |  |  |  |  |  |
| **Social Simulation** | | | | | |
| #(Intercept) | 0.4928324 | 0.0475551 | 10.363 | < 2.00E-16 | *** |
| #targett | 0.0006947 | 0.0002232 | 3.113 | 0.00201 | ** |
| #sexM | -0.2921479 | 0.0498026 | -5.866 | 1.08E-08 | *** |
|  |  |  |  |  |  |
| **Idle** | | | | | |
| #(Intercept) | 0.1766673 | 0.0337928 | 5.228 | 3.04E-07 | *** |
| #targett | 0.0004542 | 0.0001586 | 2.864 | 0.00445 | ** |
| #sexM | -0.027048 | 0.0353898 | -0.764 | 0.44524 |  |

**Supplementary Table 17**: T-tests of Reaction and Targeting times in relation to Video Game Motivations (Welch t-tests)

| **Variables** | | **Scores**  **Mean ± SD (N)** | | | **T (df)** | **p value** |
| --- | --- | --- | --- | --- | --- | --- |
| **Reaction Time (ms)** | | | | | | |
| Social Interaction | F | Y | 287.31 | ± 35.32 (60) | 0.28 (105) | 0.78 |
|  |  | N | 285.84 | ± 33.47 (144) |  |  |
|  | M | Y | 263.31 | ± 32.63 (76) | **-3.02 (137)** | **0.003** |
|  |  | N | 280.53 | ± 35.41 (68) |  |  |
|  | B | Y | 273.90 | ± 35.78 (136) | **-2.65 (278)** | **0.01** |
|  |  | N | 284.14 | ± 34.11 (212) |  |  |
| Stress Relief | F | Y | 284.20 | ± 32.21 (129) | -1.11 (139) | 0.27 |
|  |  | N | 289.85 | ± 36.69 (75) |  |  |
|  | M | Y | 271.32 | ± 36.85 (104) | -0.07 (87) | 0.94 |
|  |  | N | 271.76 | ± 29.80 (40) |  |  |
|  | B | Y | 278.45 | ± 34.88 (233) | -1.27 (224) | 0.20 |
|  |  | N | 283.56 | ± 35.39 (115) |  |  |
| Skill Development | F | Y | 286.80 | ± 35.22 (33) | 0.09 (44) | 0.93 |
|  |  | N | 286.18 | ± 33.80 (171) |  |  |
|  | M | Y | 260.55 | ± 29.08 (50) | **-2.99 (121)** | **0.003** |
|  |  | N | 277.23 | ± 36.51 (94) |  |  |
|  | B | Y | 270.99 | ± 34.01 (83) | **-2.79 (141)** | **0.01** |
|  |  | N | 283.00 | ± 34.98 (265) |  |  |
| Adrenaline Rush | F | Y | 288.97 | ± 27.96 (45) | 0.69 (88) | 0.49 |
|  |  | N | 285.52 | ± 35.50 (159) |  |  |
|  | M | Y | 269.35 | ± 37.33 (58) | -0.58 (113) | 0.57 |
|  |  | N | 272.85 | ± 33.37 (86) |  |  |
|  | B | Y | 277.92 | ± 34.81 (103) | -0.77 (194) | 0.44 |
|  |  | N | 281.07 | ± 35.22 (245) |  |  |
| Escape | F | Y | 285.44 | ± 31.05 (116) | -0.39 (167) | 0.69 |
|  |  | N | 287.38 | ± 37.58 (88) |  |  |
|  | M | Y | 271.31 | ± 32.18 (91) | -0.06 (92) | 0.96 |
|  |  | N | 271.67 | ± 39.54 (53) |  |  |
|  | B | Y | 279.23 | ± 32.25 (207) | -0.57 (262) | 0.57 |
|  |  | N | 281.48 | ± 38.94 (141) |  |  |
| Fantasy | F | Y | 286.43 | ± 28.50 (49) | 0.04 (99) | 0.97 |
|  |  | N | 286.23 | ± 35.58 (155) |  |  |
|  | M | Y | 268.43 | ± 32.18 (54) | -0.83 (123) | 0.41 |
|  |  | N | 273.25 | ± 36.54 (90) |  |  |
|  | B | Y | 276.99 | ± 31.65 (103) | -1.15 (219) | 0.25 |
|  |  | N | 281.46 | ± 36.41 (245) |  |  |
| Customization | F | Y | 282.99 | ± 29.19 (58) | -0.95 (127) | 0.34 |
|  |  | N | 287.58 | ± 35.67 (146) |  |  |
|  | M | Y | 262.53 | ± 28.36 (55) | **-2.61 (136)** | **0.01** |
|  |  | N | 276.95 | ± 37.54 (89) |  |  |
|  | B | Y | 273.03 | ± 30.44 (113) | **-2.82 (262)** | **0.01** |
|  |  | N | 283.55 | ± 36.68 (235) |  |  |
| Targeting Time (ms) | | | | | | |
| Social Interaction | F | Y | -138.64 | ± 100.01 (58) | **2.24 (106)** | **2.74E-02** |
|  |  | N | -173.55 | ± 98.85 (137) |  |  |
|  | M | Y | -66.29 | ± 107.56 (74) | **3.17 (136)** | **1.88E-03** |
|  |  | N | -124.07 | ± 107.69 (66) |  |  |
|  | B | Y | -98.08 | ± 109.99 (132) | **4.93 (269)** | **1.44E-06** |
|  |  | N | -157.46 | ± 104.16 (203) |  |  |
| Stress Relief | F | Y | -146.53 | ± 96.78 (125) | **3.14 (139)** | **2.07E-03** |
|  |  | N | -192.88 | ± 100.06 (70) |  |  |
|  | M | Y | -96.78 | ± 118.43 (101) | **-0.63 (90)** | **5.33E-01** |
|  |  | N | -85.11 | ± 90.11 (39) |  |  |
|  | B | Y | -124.30 | ± 109.59 (226) | **2.35 (214)** | **1.95E-02** |
|  |  | N | -154.32 | ± 109.30 (109) |  |  |
| Skill Development | F | Y | -167.72 | ± 109.78 (31) | **-0.26 (40)** | **7.99E-01** |
|  |  | N | -162.31 | ± 98.65 (164) |  |  |
|  | M | Y | - 49.10 | ± 104.61 (47) | **3.53 (95)** | **6.34E-04** |
|  |  | N | -115.98 | ± 107.93 (93) |  |  |
|  | B | Y | -96.25 | ± 121.02 (78) | **3.25 (114)** | **1.52E-03** |
|  |  | N | -145.54 | ± 104.31 (257) |  |  |

| Adrenaline Rush | F | Y | -153.71 | ± 98.37 (42) | **0.70 (67)** | **4.87E-01** |
| --- | --- | --- | --- | --- | --- | --- |
|  |  | N | -165.76 | ± 100.89 (153) |  |  |
|  | M | Y | -74.49 | ± 111.60 (57) | **1.69 (119)** | **9.44E-02** |
|  |  | N | -106.60 | ± 109.46 (83) |  |  |
|  | B | Y | -108.10 | ± 112.76 (99) | **2.77 (176)** | **6.25E-03** |
|  |  | N | -144.96 | ± 107.54 (236) |  |  |
| Escape | F | Y | -157.99 | ± 99.12 (110) | **0.82 (178)** | **4.15E-01** |
|  |  | N | -169.86 | ± 101.82 (85) |  |  |
|  | M | Y | -89.91 | ± 105.72 (89) | **0.49 (93)** | **6.25E-01** |
|  |  | N | -99.85 | ± 120.67 (51) |  |  |
|  | B | Y | -127.54 | ± 107.37 (199) | **1.30 (278)** | **1.96E-01** |
|  |  | N | -143.61 | ± 114.02 (136) |  |  |
| Fantasy | F | Y | -128.51 | ± 90.92 (47) | **2.92 (85)** | **4.49E-03** |
|  |  | N | -174.17 | ± 100.81 (148) |  |  |
|  | M | Y | -50.85 | ± 82.11 (54) | **4.09 (137)** | **7.34E-05** |
|  |  | N | -120.33 | ± 118.69 (86) |  |  |
|  | B | Y | -86.99 | ± 94.29 (101) | **5.69 (221)** | **4.01E-08** |
|  |  | N | -154.38 | ± 110.58 (234) |  |  |
| Customization | F | Y | -126.85 | ± 86.66 (57) | **3.57 (122)** | **5.16E-04** |
|  |  | N | -178.17 | ± 101.87 (138) |  |  |
|  | M | Y | -54.76 | ± 95.33 (53) | **3.49 (125)** | **6.80E-04** |
|  |  | N | -117.14 | ± 113.82 (87) |  |  |
|  | B | Y | -92.11 | ± 97.49 (110) | **5.27 (242)** | **3.07E-07** |
|  |  | N | -154.57 | ± 110.49 (225) |  |  |

**Supplementary Table 18**: Logistic Regressions of Reaction Time and Targeting Time in relation to Video Game Motivations

|  | | | | | |
| --- | --- | --- | --- | --- | --- |
| # | Estimate | Std. Error | t value | Pr(>\|t\|) | Significance |
| **Social Interactions** | | | | | |
| #(Intercept) | 0.683808 | 0.2148606 | 3.183 | 0.00159 | ** |
| #rt | -0.0013612 | 0.0007415 | -1.836 | 0.06726 |  |
| #sexM | 0.2134669 | 0.0527412 | 4.047 | 6.40E-05 | *** |
|  |  |  |  |  |  |
| **Stress Relief** | | | | | |
| #(Intercept) | 0.829311 | 0.212962 | 3.894 | 0.000118 | *** |
| #rt | -0.000688 | 0.000735 | -0.936 | 0.349889 |  |
| #sexM | 0.079663 | 0.052275 | 1.524 | 0.128446 |  |
|  |  |  |  |  |  |
| **Skill Development** | | | | | |
| #(Intercept) | 0.5312739 | 0.188471 | 2.819 | 0.005098 | ** |
| #rt | -0.0012907 | 0.0006505 | -1.984 | 0.048007 | * |
| #sexM | 0.16631 | 0.0462635 | 3.595 | 0.000372 | *** |
|  |  |  |  |  |  |
| **Adrenaline Rush** | | | | | |
| #(Intercept) | 2.21E-01 | 2.04E-01 | 1.083 | 0.279532 |  |
| #rt | -4.95E-07 | 7.03E-04 | -0.001 | 0.999439 |  |
| #sexM | 1.82E-01 | 5.00E-02 | 3.642 | 0.000312 | *** |
|  |  |  |  |  |  |
| **Escape** | | | | | |
| #(Intercept) | 0.6452896 | 0.2230483 | 2.893 | 0.00406 | ** |
| #rt | -0.0002678 | 0.0007698 | -0.348 | 0.72815 |  |
| #sexM | 0.0593445 | 0.0547511 | 1.084 | 0.27917 |  |
|  |  |  |  |  |  |
| **Fantasy** | | | | | |
| #(Intercept) | 0.3490366 | 0.205559 | 1.698 | 0.0904 |  |
| #rt | -0.0003802 | 0.0007094 | -0.536 | 0.5924 |  |
| #sexM | 0.1291639 | 0.050458 | 2.56 | 0.0109 | * |
|  |  |  |  |  |  |
| **Customization** | | | | | |
| #(Intercept) | 0.761419 | 0.2104952 | 3.617 | 0.000342 | *** |
| #rt | -0.0016666 | 0.0007265 | -2.294 | 0.022385 | * |
| #sexM | 0.0729077 | 0.0516697 | 1.411 | 0.159135 |  |
|  |  |  |  |  |  |
| Targeting Time and Video Game Motivations | | | | | |
| **Social Interaction** | | | | | |
| #(Intercept) | 0.4505742 | 0.0519942 | 8.666 | < 2.00E-16 | *** |
| #targett | 0.0009385 | 0.000244 | 3.847 | 0.000144 | *** |
| #sexM | 0.1657771 | 0.0544514 | 3.044 | 0.002517 | ** |
|  |  |  |  |  |  |
| **Stress Relief** | | | | | |
| #(Intercept) | 0.7190379 | 0.0519089 | 13.852 | <2e-16 | *** |
| #targett | 0.0004781 | 0.0002436 | 1.963 | 0.0505 | * |
| #sexM | 0.0471078 | 0.0543621 | 0.867 | 0.3868 |  |
|  |  |  |  |  |  |
| **Skill Development** | | | | | |
| #(Intercept) | 0.2456831 | 0.0458308 | 5.361 | 1.56E-07 | *** |
| #targett | 0.0005314 | 0.0002151 | 2.471 | 0.01398 | * |
| #sexM | 0.1397332 | 0.0479968 | 2.911 | 0.00384 | ** |
|  |  |  |  |  |  |
| **Adrenaline Rush** | | | | | |
| #(Intercept) | 0.2814196 | 0.0496955 | 5.663 | 3.23E-08 | *** |
| #targett | 0.0004047 | 0.0002332 | 1.735 | 0.0836 |  |
| #sexM | 0.1635749 | 0.0520441 | 3.143 | 0.00182 | ** |
|  |  |  |  |  |  |
| **Escape** | | | | | |
| #(Intercept) | 0.6037999 | 0.0547046 | 11.037 | <2e-16 | *** |
| #targett | 0.0002433 | 0.0002567 | 0.948 | 0.344 |  |
| #sexM | 0.0546692 | 0.0572899 | 0.954 | 0.341 |  |
|  |  |  |  |  |  |
| **Fantasy** | | | | | |
| #(Intercept) | 0.4162246 | 0.0491185 | 8.474 | 7.84E-16 | *** |
| #targett | 0.0010737 | 0.0002305 | 4.658 | 4.62E-06 | *** |
| #sexM | 0.0699149 | 0.0514398 | 1.359 | 0.175 |  |
|  |  |  |  |  |  |
| **Customization** | | | | | |
| #(Intercept) | 0.4760261 | 0.0506196 | 9.404 | < 2.00E-16 | *** |
| #targett | 0.001126 | 0.0002375 | 4.74 | 3.18E-06 | *** |
| #sexM | 0.007854 | 0.053012 | 0.148 | 0.882 |  |

* < denotes significance at <0.05

** < denotes significance at < 0.01

*** denotes significance at < 0.001
